# The impact of reduced routine community mental healthcare on people from minority ethnic groups during the COVID-19 pandemic: qualitative study of stakeholder perspectives

**DOI:** 10.1192/bjp.2024.11

**Published:** 2024-05

**Authors:** Catherine Winsper, Rahul Bhattacharya, Kamaldeep Bhui, Graeme Currie, Dawn Edge, David Ellard, Donna Franklin, Paramjit Gill, Steve Gilbert, Noreen Khan, Robin Miller, Zahra Motala, Vanessa Pinfold, Harbinder Sandhu, Swaran P. Singh, Scott Weich, Domenico Giacco

**Affiliations:** Department of Research and Innovation, Coventry and Warwickshire Partnership NHS Trust, Coventry, UK; Trust Headquarters, East London NHS Foundation Trust, London, UK; Department of Psychiatry, Medical Sciences Division, University of Oxford, Oxford, UK; Warwick Business School, University of Warwick, Coventry, UK; Division of Psychology & Mental Health, University of Manchester, Manchester, UK; Warwick Clinical Trials Unit, Warwick Medical School, University of Warwick, Coventry, UK; School of Health Sciences, Institute of Mental Health, University of Nottingham, Nottingham, UK; Department of Health Sciences, Warwick Medical School, University of Warwick, Coventry, UK; Steve Gilbert Consulting, Birmingham, UK; School of Social Policy, University of Birmingham, Birmingham, UK; Department of Sociology, University of Manchester, Manchester, UK; McPin Foundation, London, UK; Department of Research and Innovation, Coventry and Warwickshire Partnership NHS Trust, Coventry, UK; and Department of Health Sciences, Warwick Medical School, University of Warwick, Coventry, UK; School of Health and Related Research, University of Sheffield, Sheffield, UK

**Keywords:** Minority ethnic, qualitative research, COVID-19, mental health services, stigma and discrimination

## Abstract

**Background:**

Enduring ethnic inequalities exist in mental healthcare. The COVID-19 pandemic has widened these.

**Aims:**

To explore stakeholder perspectives on how the COVID-19 pandemic has increased ethnic inequalities in mental healthcare.

**Method:**

A qualitative interview study of four areas in England with 34 patients, 15 carers and 39 mental health professionals from National Health Service (NHS) and community organisations (July 2021 to July 2022). Framework analysis was used to develop a logic model of inter-relationships between pre-pandemic barriers and COVID-19 impacts.

**Results:**

Impacts were largely similar across sites, with some small variations (e.g. positive service impacts of higher ethnic diversity in area 2). Pre-pandemic barriers at individual level included mistrust and thus avoidance of services and at a service level included the dominance of a monocultural model, leading to poor communication, disengagement and alienation. During the pandemic remote service delivery, closure of community organisations and media scapegoating exacerbated existing barriers by worsening alienation and communication barriers, fuelling prejudice and division, and increasing mistrust in services. Some minority ethnic patients reported positive developments, experiencing empowerment through self-determination and creative activities.

**Conclusions:**

During the COVID-19 pandemic some patients showed resilience and developed adaptations that could be nurtured by services. However, there has been a reduction in the availability of group-specific NHS and third-sector services in the community, exacerbating pre-existing barriers. As these developments are likely to have long-term consequences for minority ethnic groups’ engagement with mental healthcare, they need to be addressed as a priority by the NHS and its partners.

Enduring inequalities in mental healthcare exist between UK minority ethnic and White British groups.^1^ Individuals from minority ethnic groups are more likely to be detained under the Mental Health Act and receive restrictive interventions.^2^ Failure to discuss cultural or religious factors, or provide accessible information for informed consent on treatment contributes to poor experiences of care. Those poor experiences, together with cultural stigma and fear of being discriminated against, generate barriers to access.^3^

During the COVID-19 pandemic people from minority ethnic groups experienced a disproportionately high impact on their mental health,^4–6^ but the reasons for this were not fully clarified. Previous literature has attributed the lack of progress in addressing ethnic inequalities in mental healthcare^7^ to inadequate understanding of the key drivers of inequalities and, in particular, the role of societal factors such as racism.^8^ The COVID-19 pandemic and resulting service changes offered an opportunity to explore which service- and societal-level factors might be involved in driving inequalities. The aim of this study was to develop a multi-level understanding of how ethnic inequalities are created and sustained in mental healthcare, drawing together the complexity of experiences from diverse ethnic and stakeholder backgrounds.^9,10^ In the current study, we focus on barriers to mental healthcare. For solutions to these barriers, the reader is directed to our companion study on improving mental healthcare through co-designed action plans.^11^

## Method

This semi-structured interview study was part of a multi-site experience-based co-design (EBCD) project to develop actions for improving access and experience of mental healthcare for people from minority ethnic groups.^11^ Study sites included four different geographical areas covered by National Health Service (NHS) mental health trusts (i.e. Coventry and Warwickshire; Greater Manchester; East London; and Sheffield). Areas were selected to reflect diversity across England, including differences in urbanicity/rurality, deprivation and ethnic composition.

Topic guides covered: (a) stakeholder perspectives on barriers to mental healthcare for minority ethnic patients prior to the COVID-19 pandemic; and (b) experiences of mental healthcare during the COVID-19 pandemic. Four trained researchers (three psychologists: C.W., N.K. and research volunteer Emily Paquini; one peer researcher: D.F.) conducted one-to-interviews, which were audio-recorded and transcribed verbatim. Patients and carers were given a £20 voucher for participating. Interviews were conducted between 8 July 2021 and 15 July 2022, following the launch of the COVID-19 vaccination programme in December 2020 and the final UK national lockdown in March 2021. Owing to continuing local restrictions and health and safety concerns, most interviews were conducted remotely (58 online interviews; 28 telephone interviews; 2 in-person interviews). The team worked closely with local clinical studies officers to ensure that participants without internet access (or those who did not want to be interviewed online) were offered an in-person or telephone interview. Researchers provided additional support to participants who had internet access but were unsure how to join online meetings.

### Patient and public involvement (PPI)

Full details on PPI roles and responsibilities are given in Supplementary Table 1, available at https://dx.doi.org/10.1192/bjp.2024.11 for . The lived experience advisory panel (LEAP) included members from each study site. The panel met online six times to provide input on ethical, recruitment, procedural and acceptability issues, in addition to views on emerging research findings. Two peer researchers were included in the core research team and contributed to all aspects of the study, including recruitment, interviewing, co-facilitating focus groups and workshops, analysis and dissemination.

### Participants

Patients were eligible if they were ≥18 years, from a minority ethnic group, had used secondary mental health services in the previous 5 years, had experienced a severe mental illness and lived in a study area. Carers were eligible if they were ≥18 years, had supported a patient (from a minority ethnic group) who had used mental health services in the previous 5 years and lived in a study area. Professionals (of any ethnicity) were eligible if they were an NHS clinician or senior manager, a community or voluntary sector worker, or a commissioner and worked in a study area.

We used purposive sampling^12^ to include diverse experiences and views from different services (e.g. drug and alcohol services, liaison psychiatry) and roles (e.g. psychologists, psychiatrists, occupational therapists) within and outside of (e.g. charity organisations) the NHS. Written (or verbal) informed consent was obtained from all participants. We conducted interviews with 88 participants (34 patients, 15 carers and 39 professionals); 87 interviews were suitable for analysis (Table 1).

**Table 1:** Summary of interviewee characteristics by site^a^ (ethnicities and diagnoses are self-reported)

Site	Patients	Carers	Professionals	Total interviews analysed
Coventry and Warwickshire	8 (2 female; 6 male) 5 Black African, 1 Somalian, 1 Indian, 1 Pakistani Diagnoses: psychosis (*n* = 5); psychosis/bipolar disorder (*n* = 1); bipolar disorder (*n* = 1); bipolar/anxiety disorder (*n* = 1)	2 (2 female) 1 Black African, 1 Pakistani	11 (8 female; 3 male) 2 Black African, 1 Black, 2 Pakistani, 1 Asian, 1 Indian, 4 White British	21
East London	8 (4 female; 4 male) 1 Caribbean, 2 Bangladeshi, 2 African; 1 East African, 1 Indian, 1 Asian Diagnoses: PTSD/anxiety (*n* = 1); PTSD/ psychosis/depression (*n* = 1); schizophrenia (*n* = 2); depression (*n* = 1); schizoaffective disorder (*n* = 1); depression/anxiety/paranoia (*n* = 1); anxiety (*n* = 1)	4 (4 female) 1 Asian, 1 Pakistani, 1 Bangladeshi, 1 Indian	10 (4 female; 6 male) 1 White Other, 3 White British, 1 Asian-mixed, 1 Indian, 1 Chinese, 2 Black, Black African	22
Greater Manchester	6 (3 female; 3 male) 2 Black, 2 Pakistani, 1 African, 1 Euro-Caribbean Diagnoses: paranoid schizophrenia (*n* = 3); schizophrenia (*n* = 1); bipolar (*n* = 1); personality disorder/bipolar disorder (*n* = 1)	4 (3 female; 1 male) 2 Asian, 1 African, 1 Pakistani	11 (7 female; 4 male) 2 White British; 1 White Mixed, 1 Asian, 1 Pakistani, 1 Black, 1 Black African, 2 mixed White Caribbean	21
Sheffield	11 (7 female; 4 male) 3 Black, 1 Indian, 2 African, 2 African Caribbean, 2 Pakistani, 1 Black Caribbean Diagnoses: psychosis (*n* = 2); bipolar (*n* = 1); schizophrenia (*n* = 3); borderline personality disorder traits (*n* = 1); not reported (*n* = 3)	5 (5 female) 1 Asian, 1 Arabic, 2 mixed Black & White Caribbean, 1 Black	7 (4 female; 3 male) 3 White British, 3 Pakistani, 1 Black	23
**Total**	**33**	**15**	**39**	**87**

PTSD, post-traumatic stress disorder.

a. Age range at each study site: site 1, 23–60 years; site 2, 29–66 years; site 3, 24–69 years; site 4, 24–62 years.

### Use of terminology

Per government guidelines,^13^ we selected the term ‘minority ethnic’, accepting that preferences in terminology vary, that ‘minority ethnic’ is not a homogeneous group and that we would not be able to include participants from all ethnicities included under this umbrella term.

### Ethics statement

The authors assert that all procedures contributing to this work comply with the ethical standards of the relevant national and institutional committees on human experimentation and with the Helsinki Declaration of 1975, as revised in 2008. All procedures involving human subjects/patients were approved by the Health Research Authority and the Health and Care Research Wales (ref. 21/WA/0181).

### Analysis

**Fig. 1:**
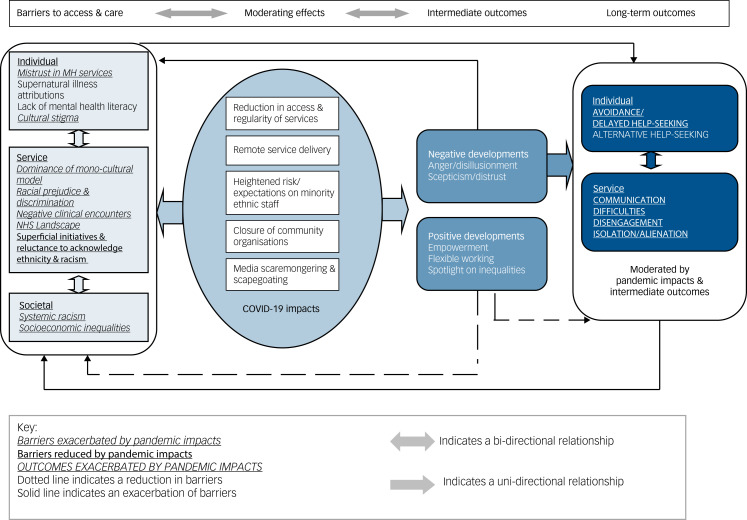
Barriers to access and mental healthcare for people from minority ethnic groups in England during the COVID-19 pandemic: a logic model of stakeholder-reported barriers, moderating effects of the pandemic, and intermediate and longer-term outcomes. MH, mental health; NHS, National Health Service.

The six stages of the framework analysis^14^ are described in Supplementary Table 2. Following development of the initial codebook by the core research team (D.G., N.K., C.W., D.F., Z.M.), C.W. conducted the analysis in NVivo 12 Pro for Windows.^15^ We selected the framework approach as it facilitates analysis of data from a large number of participants in a rigorous, transparent and logical process^16^ and enabled comparison of responses from different stakeholders and areas using a framework matrix (i.e. a grid organising each participant by row and each sub-theme by column).^17^ Using the constant comparative method, we were able to make comparisons across cases to refine each theme while tracking and grounding findings and interpretations within the raw data.^17^ During the framework process, we systematically reduced data from emergent themes to mapped impacts and outcomes (Supplementary Fig. 1). The final mapped themes and sub-themes were used to populate the logic model (Fig. 1) charting how COVID-19 impacts exacerbated pre-pandemic barriers and their outcomes. The main themes and logic model were reviewed by the research team (including peer researchers, lived experience advisors, and experts in mental healthcare, qualitative methodologies, and behavioural and organisational sciences).

## Results

Using themes and sub-themes derived from our framework analysis, we mapped out a logic model (Fig. 1) to summarise how ethnic inequalities in mental healthcare were affected by the COVID-19 pandemic. From left to right, the model delineates pre-pandemic barriers, moderating effects of the pandemic, and intermediate and long-term outcomes. As indicated in the model, hypothesised inter-relationships were complex and bi-directional, some exacerbating and others reducing barriers.

### Barriers to access and mental healthcare prior to the COVID-19 pandemic

Pre-pandemic barriers (individual, service and societal) are summarised in Fig. 1. Supplementary Table 3 presents comparative quotations from patients/carers and professionals. Supplementary Table 5 presents comparative quotations from the four different sites.

Individual-level barriers (relating to cultural background, norms and beliefs) included mistrust of mental health services, supernatural illness attributions, lack of mental health literacy and cultural stigma. Patients/carers (sites 2, 3, 4) described mistrust in services, which was attributed to previous negative experiences and/or concerns of being ‘locked away’ or over-medicated. Professionals (all sites) similarly noted a mistrust of mental health services within Roma, asylum-seeking, South Asian and Black communities, which prevented people from accessing services.

Supernatural illness attributions (e.g. black magic, jinn possession, voodoo) were commonly reported in South Asian and Black African communities by professionals (all sites) and patients/carers (sites 2, 3, 4). Supernatural beliefs led to a search for ‘other answers’ through seeking alternative remedies (e.g. exorcisms).

Patients/carers (sites 1, 2, 4) described a lack of awareness or understanding of mental ill health, which led to self-medicating (e.g. through alcohol) and delayed help-seeking. Professionals (all sites) noted a lack of mental health literacy in Chinese, South Asian and Black communities, reducing likelihood that they would access mental health services.

Cultural stigma attached to mental illness and the use of mental health services was discussed across all sites and participant types and was considered a powerful deterrent to seeking mental healthcare in South Asian, Black African, Roma and orthodox Jewish communities.

Service-level barriers included dominance of a monocultural model, racial prejudice and discrimination, negative clinical encounters, NHS landscape (e.g. competing work pressures), superficial attempts to reduce inequalities, and reluctance to acknowledge ethnicity and racism in mental healthcare. These barriers led to poor communication, disengagement from services and alienation.

Patients/carers and professionals (all sites) noted the dominance of a monocultural model. Participants reported that mental health services lacked inclusivity in terms of staff diversity (e.g. multicultural therapists), cultural understandings (e.g. intersection between ethnicity and mental illness), materials (e.g. leaflets in different languages), provisions (e.g. food choices on ward) and treatment options (e.g. ‘Tree of Life’ narrative therapy model). Linguistic and conceptual (e.g. understandings of mental illness) communication barriers were common. Some professionals (*n* = 4) from site 2 felt that they worked in ‘very diverse teams’, which facilitated communication and understanding.

Patients/carers and professionals (all sites) recalled examples of racial prejudice, stereotyping and discrimination. Patients recollected being ‘restrained a bit too often’. Carers recalled loved ones being unfairly judged or treated and ‘quickly diagnosed, misdiagnosed’. Negative verbal interactions were experienced as ‘life changing’ and included ‘snapping for no reason’, ‘microaggressions’ and ‘gaslighting’. Some patients/carers (all sites) did not report any experiences of prejudice or racism.

Negative clinical encounters were a recurring theme in patient/carer interviews (all sites). Although not explicitly linked to ethnicity, they were considered an additional barrier to help-seeking and engagement. Experiences included dismissive, insensitive and patronising attitudes, poor communication and feelings of being judged. Patients/carers noted that NHS services were ‘oversubscribed’, thus staff do not have time to ‘probe’ and that once ‘[you've had] a few years of psychology [your] quota's gone’. Professionals (all sites) concurred that services were under-resourced, making the provision of culturally appropriate mental healthcare more challenging.

Participants noted superficial attempts at tackling inequalities (sites 2, 3, 4) and a reluctance to acknowledge ethnicity, racism and mental illness (all sites). Patients felt there was a ‘lot of talk’ but no action (sites 2, 3, 4), and professionals (all sites) noted that NHS initiatives were often a ‘tick box exercise’. Professionals felt that responsibilities were placed on minority ethnic staff to solve the problems of the system and boost the ‘corporate image’ (sites 2, 3). Patients felt that racism within services was not acknowledged (site 3) and that they were unable to speak openly about their ‘racial struggles’ for fear of being misunderstood or getting ‘shut down’ (site 4). Professionals noted a ‘lot of defensiveness’ and ‘fear of getting it wrong’, especially among White professionals, contributing to the maintenance of ‘issues about racism and discrimination’.

Societal-level barriers included socioeconomic inequalities and systemic racism. Patients (sites 3, 4) felt that their economic status contributed to judgement and discrimination within services, and professionals noted the role of financial disadvantage in the development and treatment of mental illness. Patients/carers (sites 2, 3, 4) reflected on how racism in society had had an impact on their perceptions (e.g. ‘I took [a] strong reaction to something that could've not been racial’) and behaviour (e.g. ‘[I] keep myself to myself’) within services. Professionals (sites 1, 3, 4) felt that structural racism could lead to ‘a cycle of anxiety’ or avoidance of services.

### Impacts of the COVID-19 pandemic on mental health services and patients

Impacts of the COVID-19 pandemic are summarised in Fig. 1. Ripple effects (aggravating pre-existing barriers and outcomes) included anger and disillusionment, scepticism and distrust, isolation, exacerbation of communication difficulties, and racism and division. Supplementary Table 4 presents illustrative quotations by participant type. Supplementary Table 6 presents comparable quotations by site. In the quotations presented below, participant details include stakeholder type (professional role, patient or carer), participant number, ethnicity and site.

#### Reduction in access and regularity of mental health services

Reductions in accessibility and regularity of mental health services were described by patients/carers and professionals (all sites). Patients/carers noted that services went ‘completely silent’ and were ‘less responsive’, creating an additional barrier to access, although some patients viewed the pandemic as ‘just another time of something new I had to adapt to’.

Professionals noted that COVID-19 had an impact on all patients (including White British), but ‘became more noticeable’ for some people from minority ethnic communities because there was ‘already a lack of resources for them’. Professionals (sites 2, 3, 4) described increased difficulties in obtaining interpreters, which exacerbated communication barriers for patients who could not speak English. As services became less proactive, ethnic minority patients who were unable to advocate for themselves were more likely to be ‘missed and forgotten’:

‘Coming back to communication in the way that we keep in touch with people and how people access us – I think it […] must have had a disproportionate effect on people from minority ethnic communities – because we're being less persistent in the way that we access people and support people.’ (Social worker, P16, White British, site 1)

#### Remote service delivery

Most patients/carers (all sites) from minority ethnic groups preferred face-to-face interactions; however, some liked the convenience of remote consultations. Professionals noted that remote consultations could be more convenient and cost-effective for some patients (including from minority ethnic groups: sites 1, 2) and had led to a reduction in missed appointments (sites 2, 4). However, others highlighted the ‘digital divide’ (sites 1, 2, 3) and observed that remote consultations ‘were not a productive way’ to develop trust with patients from minority ethnic groups (sites 3, 4), especially those who ‘struggle with English’:

‘This has been an issue during the pandemic because if we're doing, for example, video consultations, it's very difficult to include interpreters and often I think patients may lose patience or some may lose patience to set up these video calls, so I would say that maybe with this group of people who are not English speakers, communication can be quite challenging.’ (Consultant perinatal psychiatrist, P3, White Mixed, site 2)

#### Heightened risk and expectations on minority ethnic staff

Patients/carers (all sites) described increased pressure on mental health professionals but were not aware of differential impacts on minority ethnic staff.

Professionals from minority ethnic backgrounds (site 3) described being ‘hyper-anxious’ as they felt at greater risk from COVID-19. Professionals (sites 2, 4) noted that minority ethnic staff were expected to work in ‘red zones’ while ‘others just stepped back’, despite the increased risk:

‘[this] has happened in the NHS for a really long time, asking Black and Brown members of staff to do the jobs that people didn't really want to do, or asking them to do overtime. At one point the group of people who died, relatively was the Filipino nurses, because in Philippine culture there's a rule around saying no when people need support. They were asking these Filipino nurses to work 12–16-hour shifts six days a week because they knew they wouldn't say no. This will have a lasting impact.’ (Clinical psychologist, P4, Black British, site 4)

#### Closure of community organisations

Patients (sites 1, 2, 4) were disappointed when community groups closed as they were unable to go to their places of worship or connect with their communities. Professionals (all sites) noted that the closure of community organisations had a disproportionate impact on people from minority ethnic groups, especially those ‘not confident with speaking English’ or asylum seekers, who often relied on third-sector organisations for culturally appropriate care:

‘So, I think COVID overall has impacted everybody, but I think maybe in terms of knowing where to go for support I wonder if it's impacted people from ethnic minorities especially if they don't speak English or are not confident with speaking English, are isolated or are asylum seekers. I think it would be a real struggle. Because most culturally appropriate services we use are third-sector, charities. So, during COVID they all stopped.’ (Social worker, P17, British Pakistani, site 1)

Professionals (sites 2, 3) noted a ‘vicious drop-out of minority groups’, who ‘backed off’ following the shut-down of community organisations:

‘We're picking up Bengali women and Somali men, but that's just a small example of […] lots of other minority groups who are kind of backing off, because they get to a stage where they need that support, and they normally get it from the community to be honest with you. And they're not, they're not getting it from their local community, not as much.’ (Service manager, P18, White British, site 2)

#### Media scaremongering and stereotyping

Patients/carers (sites 1, 2, 4) and professionals (site 3) commented on scaremongering in the news, including an ‘avalanche of statistic figures’ that ‘made you have anxiety’. Patients and professionals (sites 3, 4) noted that the first lockdown coincided with media coverage of George Floyd's murder, which ‘felt really [e]ntwined’, further increasing anxiety and fear, as it was felt that ‘we'll be the last to be looked after’ (Clinical psychologist, P4, Black British, site 4). Professionals (sites 1, 3, 4) were concerned that media coverage of the pandemic was characterised by ‘sweeping statements’ and divisive reporting ‘blaming Black and Brown people’, subsequently stoking ‘racism’ and ‘misinformation’ and preventing people from socialising and engaging with services for fear of persecution.

### Positive and negative developments from the COVID-19 pandemic

Positive and negative developments are summarised in Fig. 1. Positive developments are hypothesised to reduce pre-existing barriers and outcomes, whereas negative developments are hypothesised to exacerbate existing barriers. Illustrative quotations are given in Supplementary Tables 4 and 6 (cross-site).

#### Anger and disillusionment

Patients/carers (sites 1, 2, 3) expressed anger and frustration at not being able to access services and the lack of continuity of care during the pandemic. Professionals noted disillusionment in ethnic minority communities, who ‘didn't have the fight’ (site 1), and anger as inequalities were brought to the foreground (sites 1, 2, 4):

‘I think there is a sense that some communities, particularly I think the Black community, the Black Caribbean community, feel quite left behind and so there was quite a lot of anger and that led to some psychological feelings of isolation and of loss.’ (Founder of community interest company, P2, White British, site 4)

#### Scepticism and distrust

Patients (sites 1, 2, 3) expressed scepticism during the pandemic, including disbelief of news reports (e.g. concerning overwhelmed services) and reservations about the COVID-19 vaccinations. Professionals (all sites) noted a distrust in NHS professionals and an ‘anti-authority feeling’, especially among ethnic minority groups. It was felt that it will ‘take a long time before people can trust health systems again’:

‘And a lot of our clients, obviously, who aren't from this country believe what they read on Facebook and then that's it, they go with that. I think that has put a big gap between us, especially a wall up. For their own safety, more than anything else. And I think we're the last service that they want to engage with.’ (Recovery coordinator, P1, White, site 3)

#### Empowerment

Patients (*n* = 4) from site 2 experienced empowerment during the pandemic, including a motivation to get fit and eat well. They found solace in creative pursuits, producing ‘the best poems I've ever written’ and ‘concentrating on the garden making new things’:

‘But it's only like recently like during COVID, I decided to act upon my side effects because I thought to myself, I'm not… Thank God, you know I'm still living. Even during COVID I haven't had any COVID reaction, and I thought I'm going to tackle my side effects because I'm stable and I want to improve my flow…’ (Patient, P1, Caribbean, site 2)

Professionals (sites 2, 3) marvelled at the resilience and creativity shown by some patients, causing them to reflect on the system of care they provided.

#### Flexible approach to service provision

The pandemic brought about new ways of working, improving efficiency and convenience for some patients from minority ethnic groups. Professionals (all sites) felt that changes made during the pandemic had given them new ideas for service provision, including providing a hybrid model, ‘creative’ approaches such as ‘email therapy’ and ‘immersive’ exercises to provide a ‘more diverse way of thinking, of viewing the world’.

#### Spotlight on inequalities

All stakeholder types (patients, carers and professionals) saw the pandemic as a ‘wake-up call’ bringing to attention ‘medical racism’ and ‘disparity’ (all sites). Professionals observed more open conversations within their NHS trust (site 1) and were prompted to engage in transformation work to reduce inequalities (sites 1, 2, 4):

‘In terms of after, what changed, it also brought to the forefront the disparity. Now everything is moving towards digitisation and IT. So, most of our clients, we realised – they have phones, but they're not smartphones. So, we have been able to go and argue for more money as part of this transformation we are trying to do.’ (Occupational therapist, P14, Black British, site 2)

## Discussion

Our study showed that the pandemic disproportionally affected minority ethnic patients (especially asylum seekers and non-English speaking) in England through the exacerbation of pre-existing barriers and their outcomes. As services became less proactive, patients who could not advocate for themselves were less likely to be able to access services, increasing the likelihood of avoidance or delayed help-seeking. Remote service delivery exacerbated communication difficulties and excluded those without access to smart technologies. The closure of community organisations reduced access to culturally appropriate support, increasing withdrawal and isolation in minority ethnic groups. Media scaremongering and scapegoating contributed to disengagement and isolation by stoking blame and division and increasing mistrust in services. As reported by healthcare staff in previous studies,^18,19^ mental health professionals felt that ethnic minority staff were under greater duress during the pandemic.

Barriers to mental healthcare are maintained by superficial attempts to tackle inequalities, a reluctance to acknowledge ethnicity and racism, and structural factors (e.g. socioeconomic inequalities), highlighting the need for a multi-level approach to reducing inequalities. Although patients’, carers’ and professionals’ viewpoints largely converged, negative clinical encounters were a more prominent concern for patients/carers, who described how dismissive or judgemental interactions led to distress and disengagement from services. Moving forward, safe and equitable person-centred care should include full consideration of the lived experience of minority ethnic patients,^8^ including acknowledgement of the intersection between ethnicity, racism and mental ill health.^20^ Views on barriers and COVID-19 impacts were remarkably similar across all four sites, indicating that barriers are endemic in England. However, there were some indications that area 2 is ‘leading the way’ in transformational work, with higher levels of team diversity and patient empowerment.

As the country recovers from the pandemic, it will be key to recognise the importance of third-sector organisations in providing culturally appropriate mental healthcare,^8^ including how these services can be integrated into the reshaping of the care system.^21^ The onus is on services to promote equity over simplistic views of equality, ensuring representation of marginalised groups in NHS services at all levels.^18^ Positive developments observed during the pandemic could be leveraged, including a prioritisation of empowering, recovery-oriented models,^22^ incorporating nature and creative therapies as anti-oppressive approaches.^8,23^ Adopting a hybrid model to service delivery might help improve access for some minority ethnic patients,^24^ including those concerned about the stigma associated with visiting services. The heightened focus on ethnic inequalities as a result of the pandemic might help services argue for more resources (e.g. to reduce digital exclusion^5^) and encourage open conversations about racism,^25^ ethnicity and intersections with mental ill health.

### Limitations

We used a logic model to organise and present our qualitative findings. This enabled us to conceptualise (and visually depict) pre-pandemic barriers to access and care, and potential COVID-19 impacts that might have moderated these barriers and their outcomes. However, it should be noted that this was not a full logic model analysis. Rather, we used the logic model to allow a depiction of the themes and sub-themes that emerged from our framework analysis, i.e. we used an inductive approach to fully capture our stakeholders’ views and experiences, rather than imposing an *a priori* theoretical framework on the analysis. Future research might aim to elucidate the mechanisms underpinning the inter-relationships between impacts and barriers/outcomes to inform innovations in equity-driven mental healthcare.

Our purposive sampling approach might have led to the exclusion of some groups, e.g. those with less severe mental illness who did not use secondary care services. Most patient/carer participants identified as having Black or South Asian heritage and were recruited in urban areas. The extent to which findings are generalisable to other ethnic backgrounds (e.g. Romany, Chinese) or to rural areas is unclear. Despite our best efforts, we were unable to recruit non-English speaking participants, a group that professionals indicated were especially affected by the impacts of the pandemic. Most interviews were conducted virtually. This might have hindered some conversations (e.g. affected the building of trust), but also increased access and convenience for some participants. We were unable to include all impacts of the COVID-19 pandemic (e.g. increased financial disadvantage) in our analysis owing to space limitations.

### Future directions

The COVID-19 pandemic appears to have exacerbated ethnic inequalities in mental healthcare and more broadly. Some positive developments were also reported, which need to be actively pursued by services, including a focus on recovery-oriented treatment options and equity. However, the reduction of community support through NHS and third-sector services has resulted in experiences of discrimination and poor communication with services, fuelling alienation, prejudice and mistrust. What happened during the COVID-19 pandemic may have a long-lasting impact on the access and engagement of people within minority ethnic groups, particularly those who are most vulnerable and marginalised. To offset or reduce negative consequences, the NHS and integrated care partnerships will need to address them promptly and radically. This could be helped by learning from patients’ voices to further transformative models for community care.

## Supporting information

Winsper et al. supplementary material Winsper et al. supplementary material

## Data Availability

Requests for the data that support the findings of this study should be directed to the corresponding author, D.G., who will discuss them with the lead institution, Coventry and Warwickshire Partnership NHS Trust.
