# The impact of routine community mental health care reduction on people from minority ethnic groups during the COVID-19 pandemic: qualitative study of stakeholder perspectives

**DOI:** 10.1192/bjp.2024.11

**Published:** 2024-05

**Authors:** Catherine Winsper, Rahul Bhattacharya, Kamaldeep Bhui, Graeme Currie, Dawn Edge, David Ellard, Donna Franklin, Paramjit Gill, Steve Gilbert, Noreen Khan, Robin Miller, Zahra Motala, Vanessa Pinfold, Harbinder Sandhu, Swaran P Singh, Scott Weich, Domenico Giacco

**Affiliations:** 1 Research and Innovation, Caludon Centre, Coventry and Warwickshire Partnership Trust, Clifford Bridge Road, Coventry, CV2 2TE; 2 East London NHS Foundation Trust, Robert Dolan House, Trust Headquarters, 9 Alie Street, London, E1 8DE; 3 Department of Psychiatry, Medical Sciences Division, University of Oxford, OX1 2JD; 4 Warwick Business School, University of Warwick, Coventry, CV4 7AL; 5 G6 Coupland 1 Building, The University of Manchester, M13 9PL Manchester; 6 Warwick Clinical Trials Unit, Warwick Medical School, University of Warwick, Coventry, CV4 7AL; 7 School of Health Sciences, Institute of Mental Health, University of Nottingham, Nottingham, UK; 8 Health Sciences, Warwick Medical School, University of Warwick, Coventry, CV4 7AL; 9 Steve Gilbert Consulting, Suite 2A, Blackthorn House, St Pauls Square, Birmingham B3 1RL; 10 School of Social Policy, University of Birmingham, Edgbaston, Birmingham, B15 2TT; 11 Department of Sociology, University of Manchester, Manchester, UK; 12 The McPin Foundation, 7-14 Great Dover Street, London, SE1 4YR; 13 School of Health and Related Research, University of Sheffield, The University of Sheffield, Western Bank, Sheffield, S10 2TN

## Abstract

**Background:**

Enduring ethnic inequalities exist in mental healthcare. The Covid-19 pandemic has widened these.

**Aims:**

To explore stakeholder perspectives on how the Covid-19 pandemic has increased ethnic inequalities in mental healthcare.

**Method:**

A qualitative interview study of four areas in England with 34 service users, 15 carers and 39 mental health professionals from NHS and community organisations (July 2021-July 2022). Framework analysis was used to develop a logic model of inter-relationships between pre-pandemic barriers and Covid-19 impacts.

**Results:**

Impacts were largely similar across sites with some small variations (e.g., positive service impacts of higher ethnic diversity in area 2). Pre-pandemic barriers at individual level included mistrust and thus avoidance of services and at a service level, the dominance of a monocultural model, leading to poor communication, disengagement, and alienation. During the Covid-19 pandemic remote service delivery, closure of community organisations, and media scapegoating exacerbated existing barriers by worsening alienation and communication barriers, fueling prejudice and division, and increasing mistrust in services. Some minority ethnic service users reported positive developments, experiencing empowerment through self-determination and creative activities.

**Conclusions:**

During the Covid-19 pandemic some service users showed resilience and developed adaptations which could be nurtured by services. However, there has been a reduction in the availability of group-specific NHS and third sector services in the community exacerbating pre-existing barriers. As these developments are likely to have long term consequences for minority ethnic groups’ engagement with mental health care, they need to be addressed as a priority by the NHS and its partners.

## Introduction

Enduring inequalities in mental healthcare exist between UK minority ethnic and White British groups.^1^ Individuals from minority ethnic groups are more likely to be detained under the Mental Health Act and receive restrictive interventions.^2^ Failure to discuss cultural or religious factors, or provide accessible information for informed consent on treatment contributes to poor experiences of care. Those poor experiences, together with cultural stigma and fear of being discriminated against generate barriers to access.^3^

During the Covid-19 pandemic people from minority ethnic groups have experienced a disproportionately high impact on their mental health, ^4–6^ but the reasons for this were not fully clarified. Previous literature has attributed the lack of progress in addressing ethnic inequalities in mental healthcare ^7^ to inadequate understanding of the key drivers of inequalities, and in particular, the role of societal factors such as racism.^8^ The Covid-19 pandemic and resulting service changes offered an opportunity to explore which service and societal level factors might be involved in driving inequalities. The aim of this study was to develop a multi-level understanding of how ethnic inequalities are created and sustained in mental healthcare, drawing together the complexity of experiences from diverse ethnic and stakeholder backgrounds.^9 10^ In the current study, we focus on barriers to mental healthcare. For solutions to these barriers, the reader is directed towards our companion study on improving mental healthcare through co-designed action plans.^11^

## Method

This semi-structured interview study was part of a multi-site experience-based co-design (EBCD) project to develop actions for improving access and experience of mental healthcare for people from minority ethnic groups.^11^ Study sites included four different geographical areas covered by National Health Service mental health trusts (i.e., Coventry and Warwickshire, Greater Manchester, East London, and Sheffield). Areas were selected to reflect diversity across England including differences in urbanicity/rurality, deprivation, and ethnic composition.

Topic guides covered: 1) stakeholder perspectives on barriers to mental healthcare for minority ethnic service users prior to the Covid-19 pandemic; and 2) experiences of mental healthcare during the Covid-19 pandemic. Four trained researchers (3 psychologists: CW, NK, EP; 1 peer researcher: DF) conducted one-to-interviews, which were audio-recorded and transcribed verbatim. Service users and carers were given a £20 voucher for participating. Interviews were conducted between 8^th^ of July 2021 and 15^th^ of July 2022, following the final UK national lockdown in March 2021, and the launch of the Covid-19 vaccination programme in December 2020. Due to continuing local restrictions and health and safety concerns, most interviews were conducted remotely (online interviews=58, telephone interviews=28, in-person interviews=2). The team worked closely with local clinical studies officers to ensure that participants without internet access (or those who did not want to interview online) were offered an in-person or phone interview. Researchers provided additional support to participants who had internet access but were unsure how to join online meetings.

### Patient and Public Involvement (PPI)

Please see Supplementary Table 1 for full details on PPI roles and responsibilities. The lived experience advisory panel (LEAP) comprised members from each study site. The panel met online six times to provide input on ethical, recruitment, procedural and acceptability issues, in addition to views on emerging research findings. Two peer researchers were included in the core research team and contributed to all aspects of the study including recruitment, interviewing, co-facilitating focus groups and workshops, analysis, and dissemination.

### Participants

Service users were eligible if they were ≥ 18 years, from a minority ethnic group, had used secondary mental health services in the previous five years, had experienced a severe mental illness, and lived in a study area. Carers were eligible if they were ≥ 18 years, had supported a service user (from a minority ethnic group) who had used mental health services in the previous five years, and lived in a study area. Professionals were eligible if they were an NHS clinician or senior manager, a community or voluntary sector worker, or a commissioner (of any ethnicity) and worked in a study area.

We used purposive sampling ^12^ to include diverse experiences and views from different services (e.g., drug and alcohol service, liaison psychiatry) and roles (e.g., psychologists, psychiatrists, occupational therapists) within and outside (e.g., charity organisations) of the NHS. Written (or verbal) informed consent was obtained from all participants. We conducted interviews with 88 participants (34 service users, 15 carers and 39 professionals); 87 were suitable for analysis (Table 1).

### Use of terminology

Per Government guidelines,^13^ we selected the term ‘minority ethnic’ accepting that preferences in terminology vary, ‘minority ethnic’ is not a homogenous group, and that we would not be able to include participants from all ethnicities included under this umbrella term.

### Ethical approval

The study received ethical approval from the Health Research Authority and the Health and Care Research Wales (ref 21/WA/0181).

### Analysis

**Fig 1:**
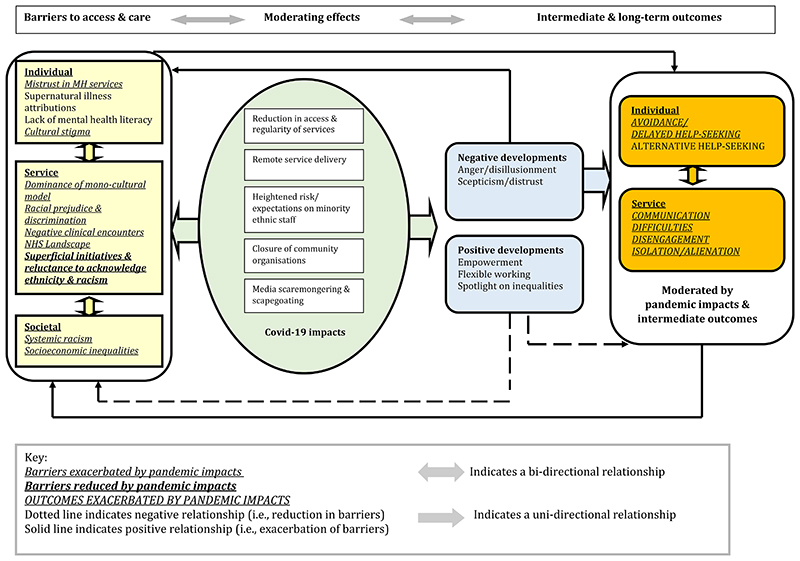
Barriers to access and mental health care for people from minority ethnic groups: A logic model of stakeholder reported barriers, moderating effects of the Covid-19 pandemic, intermediate and longer- term outcomes

The six stages of the framework analysis ^14^ are described in Supplementary Table 2. Following development of the initial codebook by the core research team (DG, NK, CW, DF, ZM), CW conducted the analysis in NVivo 12 Pro.^15^ We selected the framework approach as it facilitates analysis of data from a large number of participants in a rigorous, transparent and logical process,^16^ and enabled comparison of responses from different stakeholders and areas using a framework matrix (i.e., grid organising each participant by row and each sub-theme by column).^17^ Using the constant comparative method, we were able to make comparisons across cases to refine each theme whilst tracking and grounding findings and interpretations within the raw data.^17^ During the framework process, we systematically reduced data from emergent themes to mapped impacts and outcomes (Supplementary Fig 1). The final mapped themes and sub-themes were used to populate the logic model (Fig 1) charting how Covid-19 impacts exacerbated pre-pandemic barriers and their outcomes. The main themes and logic model were reviewed by the research team (including peer researchers, lived experience advisors, and experts in mental healthcare, qualitative methodologies, and behavioural and organisational sciences).

## Results

Using themes and sub-themes derived from our framework analysis, we mapped out a logic model (Fig 1) to summarise how ethnic inequalities in mental healthcare were impacted by the Covid-19 pandemic. From left to right the model delineates pre-pandemic barriers (yellow boxes), moderating effects of the pandemic (green ellipse), and intermediate (blue boxes) and long-term (orange boxes) outcomes. As indicated in the model, positive (i.e., in the same direction - exacerbating barriers) and negative (i.e., in the opposite direction - reducing barriers) associations.

### 1 Barriers to access and mental healthcare for people from minority ethnic groups prior to the Covid-19 pandemic

Pre-pandemic barriers (individual, service, and societal) are summarised in Fig 1. Supplementary Table 3 presents comparative quotations from service users/carers and professionals. Supplementary Table 5 presents comparative quotations from the four different sites.

Individual level barriers (relating to cultural background, norms and beliefs) included mistrust of mental health services, supernatural illness attributions, lack of mental health literacy, and cultural stigma. Service users/carers (sites 2, 3, 4) described mistrust in services, which was attributed to previous negative experiences, and/or concerns of being ‘locked away’ or over-medicated. Professionals (all sites) similarly noted a mistrust of mental health services within Roma, Asylum-seeking, South Asian, and Black communities, which prevented people from accessing services.

Supernatural illness attributions (e.g., Black magic, Jinn possession, Voodoo) were commonly reported in South Asian and Black African communities by professionals (all sites), and service users/carers (sites 2, 3, 4). Supernatural beliefs led to a search for ‘other answers’ through seeking alternative remedies (e.g, exorcisms).

Service users/carers (sites 1, 2, 4) described a lack of awareness or understanding of mental ill health, which led to self-medicating (e.g., through alcohol) and delayed help seeking. Professionals (all sites) noted a lack of mental health literacy in Chinese, South Asian, and Black communities, reducing likelihood that they would access mental health services.

Cultural stigma, attached to mental illness and the use of mental health services, was discussed across all sites and participant types and was considered a powerful deterrent to seeking mental health care in South Asian, Black African, Roma, and orthodox Jewish communities.

Service level barriers included dominance of a mono-cultural model, racial prejudice and discrimination, negative clinical encounters, NHS landscape (e.g., competing work pressures), superficial attempts to reduce inequalities, and reluctance to acknowledge ethnicity and racism in mental healthcare. These barriers led to poor communication, disengagement from services, and alienation.

Service users/carers and professionals (all sites) noted the dominance of a mono-cultural model. Mental health services lack inclusivity in terms of staff diversity (e.g., multi-cultural therapists), cultural understandings (e.g., intersection between ethnicity and mental health), materials (e.g., leaflets in different languages), provisions (e.g., food choices on ward), and treatment options (e.g., ‘Tree of Life’ model). Linguistic and conceptual (e.g., understandings of mental illness) communication barriers were common. Some professionals (n=4) from site 2 felt that they worked within ‘very diverse teams,’ which helped facilitate communication and understanding.

Service users/carers and professionals (all sites) recalled examples of racial prejudice, stereotyping and discrimination. Service users recollected being ‘restrained a bit too often.’ Carers recalled loved ones being unfairly judged or treated, and ‘quickly diagnosed, misdiagnosed.’ Negative verbal interactions were experienced as ‘life changing,’ and included ‘snapping for no reason,’ ‘microaggressions’ and ‘gaslighting.’ Some service users/carers (all sites) did not perceive any experiences of prejudice or racism.

Negative clinical encounters were a recurring theme in service user/carer interviews (all sites). Though not explicitly linked to ethnicity, they were considered an additional barrier to help-seeking and engagement. Experiences included dismissive, insensitive and patronising attitudes, poor communication and feelings of being judged. Service users/carers noted that NHS services are ‘oversubscribed,’ thus staff do not have time to ‘probe,’ and once you’ve had a ‘few years of psychology’ your ‘quota’s gone.’ Professionals (all sites) concurred that services are under-resourced, making the provision of culturally appropriate mental healthcare more challenging.

Participants noted superficial attempts at tackling inequalities (sites 2, 3, 4) and a reluctance to acknowledge ethnicity, racism and mental illness (all sites). Service users felt there was a ‘lot of talk’ but no action (sites 2, 3, 4), while professionals (all sites) noted that NHS initiatives were often a ‘tick box exercise.’ Professionals felt that responsibilities were placed on minority ethnic staff to solve the problems of the system and boost ‘corporate image’ (sites 2, 3). Service users felt that racism within services was not acknowledged (site 3) and that they were unable to openly speak about their ‘racial struggles’ for fear of being misunderstood or getting ‘shut down’ (site 4). Professionals noted a ‘lot of defensiveness,’ ‘and fear of getting it wrong,’ especially amongst White professionals, contributing to the maintenance of ‘issues about racism and discrimination.’

Societal level barriers included socio-economic inequalities and systemic racism. Service users (sites 3, 4) felt that their economic status contributed to judgement and discrimination within services, while professionals noted the role of financial disadvantage in the development and treatment of mental illness. Service users/carers (sites 2, 3, 4) reflected on how racism in society had impacted on their perceptions (e.g., ‘strong reaction) and behaviour (e.g., ‘keep myself to myself’) within services. Professionals (sites 1, 3, 4) felt that structural racism could lead to ‘a cycle of anxiety’ or avoidance of services.

### 2 Impacts of the Covid-19 pandemic on mental health services and minority ethnic service users

Impacts of the Covid-19 pandemic are summarised in Fig 1 (green ellipse). Ripple effects (aggravating pre-existing barriers and outcomes) included anger and disillusionment, scepticism and distrust (blue box), isolation, exacerbation of communication difficulties (orange box), and racism and division (yellow box). Supplementary Table 4 presents quotations by participant type. Supplementary Table 6 presents comparative quotations by site.

#### Reduction in access and regularity of mental health services

Reductions in accessibility and regularity of mental health services were described by service users/carers and professionals (all sites). Service users/carers noted that services went ‘completely silent’ and were ‘less responsive,’ creating an additional barrier to access, though some service users viewed the pandemic as ‘just another time of something new I had to adapt to.’

Professionals noted that Covid-19 impacted all service users (including White British), but ‘became more noticeable’ for some people from minority ethnic communities because there was ‘already a lack of resources for them.’ Professionals (sites 2, 3, 4) described increased difficulties in obtaining interpreters, which exacerbated communication barriers for service users who could not speak English. As services became less proactive, ethnic minority service users who were unable to advocate for themselves were more likely to be ‘missed and forgotten.’

‘Coming back to communication in the way that we keep in touch with people and how people access us. I think it therefore must have had a disproportionate effect on people from minority ethnic communities. Because we’re being less persistent in the way that we access people and support people.’ (Social worker, P16, White British, site 1)

#### Remote service delivery

Most service users/carers (all sites) from minority ethnic groups preferred face-to-face interactions; however, some liked the convenience of remote consultations. Professionals noted that remote consultations could be more convenient and cost effective for some service users (including from minority ethnic groups: sites 1, 2) and had led to a reduction in DNAs (sites 2, 4). However, others highlighted the ‘digital divide’ (sites 1, 2, 3) and observed that remote consultations ‘were not a productive way’ to develop trust with service users from minority ethnic groups (sites 3, 4), especially those who ‘struggle with English.’

‘This has been an issue during the pandemic because if we’re doing, for example video consultations, it’s very difficult to include interpreters and often I think patients may lose patience or some may lose patience to set up these video calls so I would say that maybe with this group of people who are not English speakers, communication can be quite challenging.’ (Consultant perinatal psychiatrist, P3, White Mixed, site 2)

#### Heightened risk and expectations on minority ethnic staff

Service users/carers (all sites) described increased pressure on mental health professionals but were not aware of differential impacts on minority ethnic staff. Professionals from minority ethnic backgrounds (site 3) described being ‘hyper-anxious’ as they felt at greater risk from Covid-19. Professionals (sites 2, 4) noted that minority ethnic staff were expected to work in ‘red zones’ whilst ‘others just stepped back,’ despite the increased risk.

‘Which has happened in the NHS for a really long time, asking Black and Brown members of staff to do the jobs that people didn’t really want to do, or asking them to do overtime. At one point the group of people who died, relatively was the Filipino nurses, because in Philippine culture there’s a rule around saying no when people need support. They were asking these Filipino nurses to work 12-16-hour shifts six days a week because they knew they wouldn’t say no. This will have a lasting impact.’ (Clinical psychologist, P4, Black British, site 4)

#### Closure of community organisations

Service users (sites 1, 2, 4) were disappointed when community groups closed as they were unable to go to their ‘places of worship’ or connect with their communities. Professionals (all sites) noted that the closure of community organisations had a disproportionate impact on people from minority ethnic groups especially those ‘not confident with speaking English,’ or asylum seekers who often relied on third sector organisations for culturally appropriate care.

‘So, I think Covid overall has impacted everybody, but I think maybe in terms of knowing where to go for support I wonder if it’s impacted people from ethnic minorities especially if they don’t speak English or are not confident with speaking English, are isolated or are asylum seekers. I think it would be a real struggle. Because most culturally appropriate services we use are third-sector, charities. So, during Covid they all stopped.’ (Social worker, P17, British Pakistani, site 1)

Professionals (sites 2, 3) noted a ‘vicious drop out of minority groups,’ who have ‘backed off’ following the shut-down of community organisations.

‘We’re picking up Bengali women and Somali men, but that’s just a small example of… Lots of other minority groups who are kind of backing off, because they get to a stage where they need that support, and they normally get it from the community to be honest with you. And they’re not, they’re not getting it from their local community, not as much.’ (Service manager, P18, White British, site 2)

#### Media scaremongering and stereotyping

Service users/carers (sites 1, 2, 4) and professionals (site 3) commented on scaremongering in the news including an ‘avalanche of statistic figures’ which ‘made you have anxiety.’ Service users and professionals (sites 3, 4) noted that the lockdown coincided with media coverage of George Floyd’s murder, which ‘felt really intwined’ further increasing anxiety and fear, as it was felt that ‘we’ll be the last to be looked after’ (Clinical psychologist, P4, Black British, site 4). Professionals (sites 1, 3, 4) were concerned that media coverage of the pandemic was characterised by ‘sweeping statements,’ and divisive reporting ‘blaming Black and Brown people,’ subsequently stoking ‘racism’ and ‘misinformation,’ and preventing people from socialising and engaging with services for fear of persecution.

### 3 Positive and negative developments from the Covid-19 pandemic

Positive and negative developments are summarised in Fig 1 (blue boxes). Positive developments are hypothesised to reduce pre-existing barriers and outcomes as indicated by the dotted arrows, whilst negative developments are hypothesised to exacerbate existing barriers as indicated by the solid line. Quotes are shown in Supplementary Table 4 and Supplementary Table 6 (cross-site).

#### Anger and disillusionment

Service users/carers (sites 1, 2, 3) expressed anger and frustration at not being able to access services and the lack of continuity of care during the pandemic. Professionals noted disillusionment in ethnic minority communities who ‘didn’t have the fight’ (site 1), and anger as inequalities were brought to the foreground (sites 1, 2, 4).

‘I think there is a sense that some communities, particularly I think the Black community, the Black Caribbean community feel quite left behind and so there was quite a lot of anger and that led to some psychological feelings of isolation and of loss.’ (Founder of community interest company, P2, White British, site 4)

#### Scepticism and distrust

Service users (sites 1, 2, 3) expressed scepticism during the pandemic including disbelief of news reports (e.g., concerning overrun services) and reservations about the Covid-19 vaccinations. Professionals (all sites) noted a distrust in NHS professionals and an ‘anti-authority feeling’ especially amongst ethnic minority groups. It was felt that it will ‘take a long time before people can trust health systems again.’

‘And a lot of our clients, obviously, who aren’t from this country believe what they read on Facebook and then that’s it, they go with that. I think that has put a big gap between us, especially a wall up. For their own safety, more than anything else. And I think we’re the last service that they want to engage with.’ (Recovery coordinator, P1, White; S3)

#### Empowerment

Service users (n=4) from site 2 experienced empowerment during the pandemic including a motivation to get fit and eat well. They found solace in creative pursuits producing ‘the best poems I’ve ever written’ and ‘concentrating on the garden making new things.’

‘But it’s only like recently like during Covid, I decided to act upon my side effects because I thought to myself, I’m not… Thank God, you know I’m still living. Even during Covid I haven’t had any Covid reaction, and I thought I’m going to tackle my side effects because I’m stable and I want to improve my flow…’ (SU, P1, Caribbean, site 2)

Professionals (sites 2, 3) marvelled at the resilience and creativity shown by some service users, causing them to reflect on the system of care they provided.

#### Flexible approach to service provision

The pandemic brought about new ways of working improving efficiency and convenience for some service users from minority ethnic groups. Professionals (all sites) felt that changes made during the pandemic had given them new ideas for service provision including providing a hybrid model, ‘creative’ approaches such as ‘email therapy,’ and ‘immersive’ exercises to provide a ‘more diverse way of thinking, of viewing the world.’

#### Spotlight on inequalities

The pandemic was seen as a ‘wake-up call’ bringing to attention ‘medical racism,’ and ‘disparity’ (all sites). Professionals observed more open conversations within the trust (site 1) and were prompted to engage in transformation work to reduce inequalities (sites 1, 2, 4).

In terms of after, what changed, it also brought to the forefront the disparity. Now everything is moving towards digitisation and IT. So, most of our clients, we realised - they have phones, but they’re not smartphones. So, we have been able to go and argue for more money as part of this transformation we are trying to do. (Occupational therapist, P14, Black British, site 2)

## Discussion

The pandemic has disproportionally affected minority ethnic service users (especially asylum seekers and non-English speaking) through the aggravation of pre-existing barriers and their outcomes. As services became less proactive, services users who could not advocate for themselves were less likely to be able to access services increasing likelihood of avoidance or delayed help-seeking. Remote service delivery exacerbated communication difficulties and excluded those without access to smart technologies. The closure of community organisations reduced access to culturally appropriate support, increasing withdrawal and isolation in minority ethnic groups. Media scaremongering and scapegoating contributed to disengagement and isolation by stoking blame, division, and increasing mistrust in services. As reported by healthcare staff in previous studies, ^18 19^ mental health professionals felt that ethnic minority staff were under duress during the pandemic.

Barriers to mental healthcare are maintained through superficial attempts to tackle inequalities, a reluctance to acknowledge ethnicity and racism, and structural factors (e.g., socio-economic inequalities), highlighting the need for a multi-level approach to reducing inequalities. Whilst service users, carers and professionals’ viewpoints largely converged, negative clinical encounters were a more prominent concern for service users/carers who described how dismissive or judgmental interactions led to distress and disengagement from services. Moving forward, safe and equitable person-centred care should include full consideration of the lived experience of minority ethnic service users, ^8^ including acknowledgement of the intersection between ethnicity, racism, and mental ill health.^20^ Views on barriers and Covid-19 impacts were remarkably similar across all four sites, indicating that barriers are endemic in England. However, there were some indications that area 2 is ‘leading the way’ in transformational work, with higher levels of team diversity and service user empowerment.

As the country begins to recover from the pandemic, it will be key to recognise the importance of third sector organisations in providing culturally appropriate mental healthcare ^8^ including how these services can be integrated into the re-shaping of the care system.^21^ The onus is on services to promote equity over simplistic views of equality ensuring representation of marginalised groups in NHS services at all levels.^18^ Positive developments observed during the pandemic could be leveraged including a prioritisation of empowering, recovery-oriented models,^22^ incorporating nature and creative therapies as anti-oppressive approaches.^8 23^ Adopting a hybrid model to service delivery might help improve access for some minority ethnic service users,^24^ including those concerned about the stigma associated with visiting services. The heightened focus on ethnic inequalities as a result of the pandemic might help services argue for more resources (e.g., to reduce digital exclusion^5^), and encourage upfront conversations about racism,^25^ ethnicity, and intersections with mental health.

### Limitations

We used a logic model to organise and present our qualitative findings. This enabled us to conceptualise (and visually depict) pre-pandemic barriers to access and care, and potential Covid-19 impacts which might have moderated these barriers and their outcomes. However, it should be noted that this was not a full logic model analysis. Rather, we used the logic model to allow a depiction of the themes and sub-themes that emerged from our framework analysis, i.e., we used an inductive approach to fully capture our stakeholders’ views and experiences, rather than imposing an a priori theoretical framework on the analysis. Future research might aim to elucidate the mechanisms underpinning the inter-relationships between impacts and barriers/outcomes to inform innovations in equity-driven mental healthcare.

Our purposive sampling approach might have led to the exclusion of some groups, e.g., those with less severe mental illness who did not use secondary care services. Most service user/carer participants identified as having Black or South Asian heritage and were recruited in urban areas. The extent to which findings are generalisable to other ethnic backgrounds (e.g., Romany, Chinese) or rural areas is unclear. Despite our best efforts, we were unable to recruit non-English speaking participants, who were indicated by professionals as being especially affected by the impacts of the pandemic. Most interviews were conducted virtually. This might have hindered some conversations (e.g., impacted on building trust), but also increased access and convenience for some participants. We were unable to include all impacts of the Covid-19 pandemic (e.g., increased financial disadvantage) in our analysis due to space limitations.

### Future directions

The Covid-19 pandemic appears to have exacerbated ethnic inequalities in mental healthcare, and more broadly. Some positive developments were also reported, which need to be actively pursued by services including a focus on recovery-oriented treatment options and an equity focus. However, the reduction of community support through NHS and third sector services has resulted in experiences of discrimination and poor communication with services, fueling alienation, prejudice and mistrust. What happened during the Covid-19 pandemic may have a long-lasting impact on the access and engagement of people within minority ethnic groups, particularly those who are most vulnerable and marginalised. To offset or reduce negative consequences, NHS and integrated care partnerships will need to address them promptly and radically. This could be helped by learning from service users’ voices to further transformative models for community care.

## Supplementary Material

Supplementary Materials

## Figures and Tables

**Table 1:** Summary of interviewee characteristics by site (ethnicities are self-reported)

Site	Service users	Carers	Professionals	Total interviews analysed
Coventry and Warwickshire	8 (2 Female; 6 Male) 5 Black African, 1 Somalian 1 Indian, 1 Pakistani Self-reported diagnoses: psychosis (n=5); psychosis/bi-polar disorder (n=1); bipolar disorder (n=1); bipolar/anxiety disorder (n=1)	2 (2 Female) 1 Black African, 1 Pakistani	11 (8 Female; 3 Male) 2 Black African, 1 Black, 2 Pakistani, 1 Asian, 1 Indian, 4 White British	21
East London	8 (4 Female; 4 Male) 1 Caribbean, 2 Bangladeshi, 2 African; 1 East African, 1 Indian, 1 Asian Self-reported diagnoses: PTSD/anxiety (n=1); PTSD/ psychosis/depression (n=1); schizophrenia (n=2); depression (n=1); schizoaffective disorder (n=1); depression/anxiety/ paranoia (n=1); anxiety (n=1)	4 (4 Female) 1 Asian, 1 Pakistani, 1 Bangladeshi, 1 Indian	10 (4 Female; 6 Male) 1 White Other, 3 White British, 1 Asian-mixed, 1 Indian, 1 Chinese, 2 Black, Black African	22
Greater Manchester	6 (3 Female; 3 Male) 2 Black, 2 Pakistani, 1 African, 1 Euro-Caribbean Self-reported diagnoses: paranoid schizophrenia (n=3); schizophrenia (n=1); bipolar (n=1); personality disorder/bipolar disorder (n=1)	4 (3 Female; 1 Male) 2 Asian, 1 African, 1 Pakistani	11 (7 Female; 4 Male) 2 White British; 1 White Mixed, 1 Asian, 1 Pakistani, 1 Black, 1 Black African, 2 mixed White Caribbean	21
Sheffield	11 (7 Female; 4 Male) 3 Black, 1 Indian, 2 African, 2 African Caribbean, 2 Pakistani, 1 Black Caribbean Self-reported diagnoses: psychosis (n=2); bipolar (n=1); schizophrenia (n=3); borderline personality disorder traits (n=1); not reported (n=3)	5 (5 Female) 1 Asian, 1 Arabic, 2 mixed Black & White Caribbean, 1 Black	7 (4 Female; 3 Male) 3 White British, 3 Pakistani, 1 Black	23
**Total**	**33**	**15**	**39**	**87**

Age range at each study site. Site 1 = 23 to 60 years; site 2 = 29 to 66 years; site 3 = 24 to 69 years; site 4 = 24 to 62 years

## Data Availability

Requests for data sharing agreements for secondary analyses can be directed to Prof. Giacco, ARIADNE Chief investigator at domenico.giacco@warwick.ac.uk, who will discuss them with the lead institution, Coventry and Warwickshire Partnership NHS Trust.
