# Supplementary material for: The impact of reduced routine community mental healthcare on people from minority ethnic groups during the COVID-19 pandemic: qualitative study of stakeholder perspectives
Source: Br J Psychiatry. 2024 May;224(5):150–6. doi: 10.1192/bjp.2024.11 (PMC7615860; doi:10.1192/bjp.2024.11)
Supplement: Supplementary file 1 [file S0007125024000114sup001.doc]

Supplementary Table 1. GRIPP2-SF to illustrate patient and public involvement in the ARIADNE study

Reference: Staniszewska S, Brett J, Simera I, Seers K, Mockford C, Goodlad S et al. GRIPP2 reporting checklists: tools to improve reporting of patient and public involvement in research. BMJ 2017; 358 :j3453 doi:10.1136/bmj.j3453

Section and topic	Item
**1: Aim**<br>Report the aim of the study	To understand stakeholders' experiences and views of the impact of the Covid-19 pandemic on mental healthcare for people from minority ethnic groups. To collaboratively involve experts by experience at all stages of the research project.
**2: Methods**<br>Provide a clear description of the methods used for PPI in the study	• The Lived Experience Advisory Panel (LEAP) members were selected from the four study sites on the basis of: (1) having experience of using mental health services and (2) being able to usefully contribute to the study. LEAP members were Black, Black-Caribbean, African and South Asian. The LEAP was chaired and managed by a co-applicant and peer researcher, both of whom had lived experience of mental health distress as a consequence of racism. Convening six online meetings, the LEAP provided 'critical friend' discussion regarding ethical, recruitment, procedural and acceptability issues. They also gave input on emerging research results (e.g., identified priorities for change from the interviews and focus groups). LEAP members were offered the opportunity to contribute to the development of a conference presentation, participate in the filming of the videos summarising themes from stakeholders' experiences,' and share their experiences at dissemination workshops.<br>• The peer researchers were members of the core research team, which met weekly to discuss ongoing project management (e.g., recruitment strategies, data management, etc) and emerging research findings. Peer researchers received research training (e.g., interviewing technique, focus group facilitation, data management, Point of Care Foundation EBCD training) and weekly (research) supervisions from the chief investigator and the project manager. They were employed and received regular pastoral support from the McPin Foundation. The peer researchers contributed to edits of the paper and are co-authors.
**3: Results**<br>Outcomes – Report results of PPI in the study, including both positive and negative outcomes.	PPI contributed to the study in several ways:<br>• Development of study materials (peer researchers reviewed study materials for comprehension, acceptable language use, and suitability).<br>• Collection of research data, including recruiting participants (peer researchers, LEAP), conducting one-to-one interviews (peer researchers), co-facilitating focus groups and co-design workshops (peer researchers), advice on facilitating the co-production workshops (LEAP).<br>• Coding of interview transcripts (peer researchers independently coded a proportion of the interview transcripts).<br>• Development of main themes and sub-themes from the interview data (peer researchers reviewed the initial code book for omissions and contrasting interpretations; peer researchers took part in team discussions to refine the main themes and sub-themes and reviewed the final analysis framework; the LEAP commented on emerging themes and discussed in relation to resonance with their own experiences, and potential omissions).<br>• A peer researcher presented early ARIADNE findings at the ENMESH conference.<br>• Throughout the write up phase of the manuscript, the peer researchers contributed to drafts and re-drafts.
**4: Discussions**<br>Outcomes – Comment on the extent to which PPI influenced the study overall. Describe positive and negative effects	Patient and public involvement was central to the study, and informed important aspects of each stage, as described in section 3 (above). The impact of PPI was likely related to the following factors. First, ample resources and time were planned for PPI at conception stage. Second, senior lived experience leads were a key part of the research team providing pastoral support in addition to pre-existing relationships and processes. Third, most PPI partners were involved from the beginning of the project enabling them to help shape the project. Fourth, research and

	development opportunities (section 2) were provided, in addition to pastoral support in the form of regular (weekly) supervisions.<br>There were also limitations. This was a fast paced, multi-site study. We experienced some turnover of peer researchers and LEAP members. This placed additional workload on the team, and it was challenging for the peer researchers who hadn't been involved in the project from the beginning to "catch up" with all elements of the project and obtain research passports and IT permissions. We put processes in place to ameliorate difficulties including written guidance sheets (to supplement training), regular email task reminders, and weekly supervisions (both pastoral and research). Future studies might incorporate contingency plans for PPI members' turnover.
**5: Reflections**<br>Comment critically on the study, reflecting on things that went well and those that did not, so others can learn from this experience.	The PPI in the study was embedded as far as possible. However, a key challenge was time. The two peer researchers were employed on a part-time basis to ensure some geographical representation, and there was some turnover. For the employed peer researchers this reduced time for getting to grips with the study, and for the team this meant that research governance approvals had to be repeated several times as the newly employed peer researchers joined. Future studies might consider the relative benefits of part-time versus full-time positions for peer researchers.

**Supplementary Table 2. The six stages in the framework analysis process[1]**	
1) Familiarisation with the interview data (immersion)	The senior researcher (C.W) became familiar with the data by reading through each transcript and checking accuracy against the audio-files.
2) Coding	C.W read each transcript line-by-line and applied a paraphrase or label to relevant sections, using an inductive, open coding approach. This stage underpinned the development of the initial code book. A proportion (28%) of the transcripts were independently coded by the 2 psychologist researchers (N.K coded ~10%; E.P coded ~3%), and the 2 peer researchers (Z.M coded ~10% and D.F coded ~10%). The code book was shared with the core research team and discussed at length during a core researcher team meeting to discuss any discrepancies and clarify codes.
3) Development of the working analytical framework	Using the code book as a basis, codes were grouped together in categories using a tree diagram to develop the working analytical framework organised under barriers, impacts of Covid-19 and contextual factors (e.g., societal factors)
4) Applying the analytical framework	The working analytical framework was applied by C.W indexing subsequent transcripts with the existing categories and codes. This process was conducted using the annotation function in NVivo to link each code to the relevant section in the transcript (to keep grounded in the raw data).
5) Charting data into the framework matrix	Once the working analytical framework had been applied to all transcripts, we used NVivo to generate the framework matrix. The matrix comprised one row per participant and one column per code. Participants were grouped according to their type (i.e., service user, carer, and professional) and region (i.e., sites 1, 2, 3 and 4) to facilitate comparisons across stakeholder type. We used the auto summarise function to populate the framework with illustrative quotes for each code. C.W progressed through the matrix summarising the data and highlighting pertinent quotes for easy identification.
6) Interpreting the data	We generated themes from the data set by reviewing the matrix and making connections within and between participants and categories. This process was influenced by our original research questions and by new concepts which were generated inductively from the data (e.g., unexpected positive outcomes of the Covid-19 pandemic). We used analytical memos within NVivo to track the development of themes including: a definition of the category, specific codes relating to the category, a summary of the raw data noting key quotes, discussion of any refutational quotations, and further observations.
Reflexivity	C.W, N.K, E.P (research psychologists), D.F, Z.M (peer researchers) and D.G (clinical and academic psychiatrist) discussed the initial themes from the code book, which were resolved by consensus. The wider team includes experts in mental health care, qualitative methodologies, behavioural and organisational sciences, general practice and social care, and senior lived experience advisors.
[1]Gale NK, Heath G, Cameron E, et al. (2013) Using the framework method for the analysis of qualitative data in multi-disciplinary health research. BMC medical research methodology;13(1):1-8.	

Supplementary Table 3. Barriers to mental health access and care for minority ethnic service users: subthemes and illustrative quotations organised by participant type (ethnicities are self-reported; interviews conducted across four sites covered by NHS trusts: Coventry and Warwickshire; East London; Greater Manchester; Sheffield)	
**SERVICE USER (SU)/CARER (C) (S=site)**	**PROFESSIONAL (S=site)**
**INDIVIDUAL BARRIERS**	
**Sub-theme: Mistrust of MH services**<br>English is not her first language so where she kind of (08:33) erm a lot of service users are so scared to be like sectioned or to be taken away or to be made to feel like they're ill or have to up their dose of medication they will just lie to the doctor and say things that aren't true. (C, P15, Indian; S2)	I'm someone who was an asylum seeker and a refugee, a long time ago now. We feel that systems are all working together. Then, there's also worry of not being able to trust health services, because how might they use that information against you. (Clinical psychologist, P4, Black British; S4)
**Sub-theme: Supernatural illness attributions**<br>A lot of it is the devil. At one point [name removed] was like this, he even brought two women to the home, and they burnt coals and started reading the Quran to him, to take the devil away from him. (C, P20, Arabic British; S4)	I would say, both culturally and I guess religion as well has definitely played a big part in how people understand their experiences of their mental illness. That might be because of hearing voices, and how that is interpreted. It might be seen as a weakness. I've heard mental health described as a White person illness before, Western affliction before. Or have wanted to more go down the route of again spiritual healings, natural remedies. (Occupational therapist, P4, White British; S1)
**Sub-theme: Mental health literacy**<br>She was undiagnosed for a very long time, so we didn't reach out for help because we didn't know what it was. We just thought you know that's how she is erm and we found out on erm a channel, just watching like a Channel 4 documentary and were just like "Oh my gosh." (C, P15, Indian; S2)	And I'm thinking of one particular person, and their family they have pharmacists in their family, and they were the people… in fact I had a GP trainee sometime back and their brother had symptoms for some time, and they just didn't, again, lack of understanding, and I'm just thinking you are someone who probably knows a bit more about this and it's still taking you some time and I don't know whether there's certain avoidance as well. (Consultant psychiatrist, P8, British Asian; S1)
**Sub-theme: Stigma**<br>I was finding it very hard to accept, very hard to face the people who know. I was absolutely petrified. I thought, "oh my god, what are people going to think of me, that I'm a mental case, that I'm mad. Yes, I do think that the Asian community or minority community do not get the help and support they should get and it's not a problem from the NHS but also a cultural issue. (SU, P18, British Pakistani; S1)	Because many families, they don't want…other people in the community to know that their relative is going through a mental health crisis, because there's a stigma attached to that. So, by the time they come to us, they've either done something, like take an overdose or cut themselves, and then they seek help that way. (Senior mental health liaison nurse, P17, Black British; S2)
**SERVICE LEVEL BARRIERS**	
**Sub-theme: Dominance of mono-cultural model**<br>I was having difficulties of not having a therapist that could relate in someone who is in Muslim background and who I could feel comfortable about it. Because most of the psychiatrists that I have seen and most of psychologists that I have seen are Caucasian European. The first five-six sessions I had to explain what is in Islam, the interaction of Islam with mental health. (SU, P6, East African; S2)	A lot of the publications are generally for English-speaking people. It has shown on my patients' data that it does make them feel marginalised because of the fact that it doesn't feel it's for them. (Recovery coordinator, P10, Black; S3)
**Sub-theme: Racial prejudice and discrimination**<br>I even had a problem with my psychiatrist that I've gone to hospital a couple of years ago, because I found him very, very racist…. I've seen him speaking to other people in the way he would, and then with me, he was like, very hostile and stuff like that… I know for a fact if I speak to anybody in a way… I notice they don't say much, I know if I get those sort of looks as I could have, I know exactly what it is - so I know it's because I'm Black. (SU, P2, Black; S3)	Sometimes… as minority ethnic staff members, and you can actually see people who are unwilling to work with you, who are quite guarded. And you pick up on it. You pick up – there is a difference in how this family or client relates to you. Because sometimes clients come in: 'No, I don't want to work with that staff member. I want a White doctor. (Occupational therapist, P14, Black British African; S2)
**Sub-theme: Negative clinical encounters**<br>I think I mentioned to my key worker that I wasn't feeling well, and I was told you don't look like you're not feeling well, and then I got very angry at my key worker, and I was a bit rude to her and then what's it called I completely let go of the services of the early intervention team because I thought they weren't helping me at all so what was the point of being with them. (SU, P22, Somalian; S1)	No specific responses
**Sub-theme: NHS Landscape**<br>Yeah. Not so much because the thing is there's so many women in the ward. They can't… I don't think they've got time to do all that, and then without getting personal but their way of doing it is to give you 15 to 20 tablets a day so that you don't know where you are. (SU, P3, British Pakistani; S3)	I think it's quite dispiriting for mental health teams to… they just must be exhausted and then you're just trying to survive and then the concept of doing it right for a different group is very hard when overall the NHS is pretty much on its knees really. So, I think that and the effects just of having so many different languages and cultures to try and address.' (P12, Commissioner, White British; S2)

**<u>Sub-theme: Tokenistic/superficial attempts at tackling inequalities</u>**<br>It's not being done for us for our benefit. At least that's what it feels like anyway, I feel like there's a lot of talk. Yeah. But in terms of action…(SU, P19, Black British; S4)	So, classic one was with Black Lives Matter. That was a very… I'm sorry, calling it a token is where a colleague of mine who was Black felt very upset, because you've got white managers saying, 'Well, I think you'd be interested in this, I think you should lead on this'… And, actually, it felt unpleasant, that it's not her responsibility, you know! And it seems to be a kind of… And I would use the word 'token' because I think the corporate image… (Consultant psychiatrist, P11, British Indian; S2)
**<u>Sub-theme: Reluctance to acknowledge ethnicity, racism, and mental health</u>**<br>It's because they didn't really accept the fact of it, that some people even if they are support workers, care-coordinators, psychiatrists, they don't accept in the mental health system that people still are racist and that people are discriminatory, as well. It's out in the world, it's probably always going to be there. It'd just be nice for them to say, 'Yeah, you know what, actually you know what, he was racist, you were right about it.' It's not like that… It's all ignored, nobody is bothered about it. (SU, P2, Black; S3)	I never had found anybody in my time at the NHS who is having those conversations unless you seek it out externally, through projects like Black Minds Matter which is an UK organisation. That is something, again, because as my role as an OT I speak a lot to people around their identity and their roles and how that forms who they are. And their experiences of the world. And yet, race, ethnicity, religion, and culture are key aspects of that and yet, it's a massive taboo area that nobody goes near. Because I think of our…when I say "our" I do think mostly White professionals…I don't want to get it wrong. (Occupational therapist, P4, White British; S1)
**SOCIETAL FACTORS**	
**<u>Sub-theme: Socio-economic inequalities</u>**<br>I feel like, you know, we're a very working-class family, like you know, there's not a high level of education in my family and I feel like you are sort of judged on that as well, sort of being an ethnic minority maybe being a lower level of education, you know the way you talk, the way you present yourself, the way you dress or whatever. I do feel like, if in different circumstances, maybe a different family would have got support earlier maybe. (C, P22, Black and White Caribbean; S4)	In more deprived areas, where I've worked with…in terms of, kind of in the depths of deprivation, I guess it would… inevitably but not surprisingly, there are more challenges in helping people improve with their mental health. Now in terms of…I guess that boils down to a level of education and literacy on the patient's part, finding it difficult in things around remembering medication regimes, more taking on board pieces of advice. (General practitioner, P2, British Pakistani; S1)
**<u>Sub-theme: Systemic racism</u>**<br>I thought it was racial and that's why I reacted the way I did. That's just an example of how because of like the system and society and everything, I took such a strong reaction to something that could've not been racial. (SU, P13, British Black Caribbean; S4)	I think it's also difficult when somebody has had a significant experiences of hate crime, or a racial abuse within services or within the wider world. And that is then kind of perpetuated in a cycle of anxiety for example or avoidance. (Occupational therapist, P4, White British; S1)

**Supplementary Table 4. Covid-19 impacts on services and ethnic minority service users and positive developments: Illustrative quotations organised by participant type (ethnicities are self-reported; interviews conducted across four sites covered by NHS trusts: Coventry and Warwickshire; East London; Greater Manchester; Sheffield)**	
**SERVICE USER (SU)/CARER (C) (S=site)**	**PROFESSIONAL (S=site)**
**IMPACTS OF THE COVID-19 PANDEMIC**	
**Sub-theme: Reduction in access & regularity of mental health services**<br>There's a thicker skin involved in the service than there was in respect of what, the layers of support are not there. You probably have to shout three times to get the first acknowledgement whereas before you know it was proactive. It's now reactive. (SU, P9, Euro-Caribbean; S3)	So, we weren't getting the referrals because people weren't being seen and they weren't being identified, they were just getting quietly ill which differentially then affects ethnic groups because they don't access… A lot of them don't… You know the story is that they… Barriers for them already, and there was additional barriers with COVID with things being shut and then in the August everything just went poof, you know. (Consultant Psychiatrist, P7, White British; S4)
**Sub-theme: Remote delivery of mental health services**<br>…but on the phone, it's much more easy to say that to someone, and dismiss them, because you're not doing it to their face. So in terms of switching to phone calls during the pandemic, it had an impact of, just an additional thing of not being listened to, just an additional barrier. (SU, P5, Indian Heritage; S4)	I still believe it [remote consultations] is particularly difficult for certain people from certain ethnic backgrounds. Like when you look at other determinants of pro-engagement to the services, it is worse with the Black and ethnic minority groups. Because some of them they have no access to even telephones, some of them don't have telephones. Some of them will not attend for appointments because they don't have a telephone where they can be contacted. Some of them do not know how to read or write. (Psychiatrist, P17, Black African; S3)
**Sub-theme: Heightened risk/expectations on minority ethnic staff**<br><br>No specific responses.	She was talking about, because she's not from this country, and what she observed was that, if we say… The local staff, not from those backgrounds, actually, we're more likely to not be as proactive or wanting to support clients with Covid or suspected Covid or going to red zones and things like that. The other thing to be aware particularly in my field is that predominantly the workforce is from Black-Asian and minority ethnic backgrounds, as well. So, it's a reflection from a colleague about how the minority workforce felt, that they felt that they had to go in, they had to go on the red zones, they had to that, whereas the others just stepped behind, even full on knowing that the risk was higher. (Consultant psychiatrist, P11, British Indian; S2)
**Sub-theme: Closure of community organisations**<br>So, I used to go to [community group for African Caribbeans] every week for a meal and it had to shut down because of the pandemic, so I couldn't socialise with them kind of people anymore. (SU, P8, African-Caribbean; S4)	So, we couldn't get people out to services back into their own areas where other treatment may be available - mutual aid, things like that. Because I know there is Farsi NA groups in [area removed]. And there is other cultural… I know there is Chinese NA group in [area removed] as well. But it's things like that, that unfortunately everything is just gone all at once. It's only just starting to re-open again. (Recovery Coordinator, P1, White; S3)
**Sub-theme: Media scaremongering and stereotyping**<br>And the news was all bad, this many deaths, this many of this and that, everything was really very doom and gloom, so it was quite a scary time, yeah. (C, P22, Pakistani; S2)	Because on the news there was stuff like, we need to put [area removed] on a local lockdown, because it's going to be Eid and all the borough people are going to be out and mixing with each other, versus my actual experience of that, which is not having seen family members and friends for months at a time. There was lots of things online and on TV essentially blaming Black and Brown people, versus what was actually going on in the Black and Brown community. (Clinical psychologist, P4, Black British; S4)
**Sub-theme: Scepticism and distrust**<br>And I think also (25:19), I don't watch the news so I'm not entirely sure what's going on if I'm honest. In terms of covid at the moment, I think it's still something that's dragging out with the vaccinations, I think everybody has anxiety about it, and I'm just in that boat with everybody else where, don't know what's happening with the vaccinations, and now it's those passports. (SU, P5, Indian Heritage; S4)	So, what's emerged in our area despite massive work happening, our vaccine uptake is poor. Especially amongst ethnic minority groups. Why? Well for all the reasons that you'll note but mainly disaffection and anti-authority feeling. I don't trust you. That's crippling for us that have worked here forever but it's very difficult to accept. Okay, don't trust the government, who does? but you should trust me I'm your doctor. And then realising that you're just sort of part of the system. (Commissioner, P12, White British; S2)
**Sub-theme: Anger, frustration, and disillusionment**<br>So, I think on a practical level with health professionals, where there's been no face to face, I think there can often be that irritability of 'they are not bothered, they are not available, but then because it's not the same is it? (C, P13, British Bangladeshi; S2)	Perhaps a reflection on the health inequality that already exists within our system, and I guess upset, resentment and anger around that. (Occupational Therapist, P4, White British; S1)
**POSITIVE DEVELOPMENTS FROM COVID-19**	
**Sub-theme: Empowerment**<br>I had to do a lot of the things myself. It was quite empowering because if I couldn't get through to the CMHT or to the doctors or to the nurses then I had to find my own solution, it was more self-help. I mean I found out my hobbies was restricted I couldn't go to art galleries if I wanted to or go to listen to a poetry event or go to, I used to go to a library for poetry class that was	And I also think with a number of them it was a bit empowering as well, we were having some model, I mentioned before the maps that we do in the therapy that I offer and normally I'd draw those out on a paper on a table between and obviously I couldn't do that online. But young people are creative, so they'd create the map and share it with me and so for them they were really taking ownership over their therapy. That felt really positive, that they were able to do that. (Clinical psychologist, P21,

stopped; all the libraries was closed. So, I found it was restrictive but then I found that I could find other things to do; other talents emerged from that. (SU, P7, African; S2)	Black and White Caribbean; S3)
**<u>Sub-theme: Flexible approach to service provision</u>**<br>I think… they've still had to make improvements and adapt in other areas of health because of Covid, because now when you ring the surgery, the GP practice, you can have a phone consultation instead of having to go all the way in the surgery. And sometimes that's all you need. I think because they have to adapt now to the Covid pandemic, they're trying to make other areas of health more accessible. I would say they've made it more accessible. (SU, P1, Caribbean; S2)	I think also as long as you can work out how to interact here the use of images, sounds to be more creative. Which I think is one of the things I've learnt with working with ethnic minority communities is there's a much more diverse way of thinking of viewing the world. That's really worked well. So, we will run mindfulness exercises where we're using all the five senses and you can do that within this. Much more in an immersive way, so that's a real positive, I think. (Founder of community interest group, P2, White British; S4)
**<u>Sub-theme: Spotlight on inequalities</u>**<br>Yes, and you know when I talked about institutional racism outright, they just don't want to believe it, but there is injustice, inequality for the BME community, now they are picking it up with the pandemic it's certainly been picked up hasn't it? More visual. (Carer, P19, Asian British; S2)	But there were a lot of people that were donating laptops to organisations and places, to hopefully fill those gaps and then each organisation knew their clients better, so they tried to use their ways of making sure how these people would be accessing the zoom. For example, we used to do one-to-one coffee mornings to keep them engaged because they had mental health issues and it was the nature of their wellbeing, so it was my priority to get support for those who couldn't afford it and I did request and apply, and I managed to get 19 iPads. (Mental health recovery worker, P6, Pakistani; S4)

Supplementary Table 5. Barriers to mental health access and care for minority ethnic service users: subthemes and illustrative quotations organised by site and participant type (service user/carer followed by professional; ethnicities are self-reported; interviews conducted across four sites covered by NHS trusts: Coventry and Warwickshire; East London; Greater Manchester; Sheffield)

SITE 1	SITE 2	SITE 3	SITE 4

INDIVIDUAL BARRIERS			
**Sub-theme: Mistrust of MH services**			
	• English is not her first language so where she kind of, erm, a lot of service users are so scared to be like sectioned or to be taken away or to be made to feel like they're ill or have to up their dose of medication they will just lie to the doctor and say things that aren't true. (C, P15, Indian)	• Because the things that she went through like when she was manhandled by the police, she was all bruised up and everything, it was very distressing for her after that event of them removing her from the flat, she was very wary, she was scared of the police for quite a while. [C, P22 Black & White Caribbean]	• I didn't trust the system very much. But I have been I guess since then, so it's been good. (SU, P16, Black British)
• To be honest it's vast. I worked with Roma communities where it's a big issue. But more so as a mistrust of services. (Occupational therapist, P4, White British)	• These are in historic structures that our communities don't trust and feel unsafe in. Whether that's from sectioning or not or from cancer services or other services. How can you expect people that are already uncomfortable approaching these structures to engage and to really truly be themselves authentically and to speak on their traumas or what's happening behind closed doors at home? (Charity founder, P21, Black African)	• Okay so, it's always been hard for people from ethnic backgrounds because it's about trust. But it is really hard because a lot of professionals don't. They won't believe in the Jinn. If the patient says to the CPN I've got Jinn possession, they attacked me. They won't tell them because they think they'll get sectioned, or they'll get their medication increased. (Recovery coordinator, P7, British Pakistani)<br>• **<u>Refutational view:</u>** I think from 15 years ago to now there's been a big change in how the BAME community engages with services. I think there is still, to an extent, but not as much fear or cautiousness that they used to show previously - they were quite cautious to work with services in the past. But I think, currently, it's a fairly positive one. I think they trust services… Again, I'm generalising, obviously, you'll have certain cases which aren't the case. (Social worker, P6, British Asian)	• I'm someone who was an asylum seeker and a refugee, a long time ago now. We feel that systems are all working together. Then there's also worry of not being able to trust health services, because how might they use that information against you. (Clinical psychologist, P4, Black British)
**Sub-theme: Supernatural illness attributions**			
	• I just came into the UK from Nigeria and the first psychiatrist I met, bless him, in [area removed] he said to me…he asked me a lot of questions, I was saying things about witchcraft, and…why is this telling me to do with this, and do that. (SU, P5, African)	• …especially with the Muslim community, when mental health comes in some believe that it is like evil spirits that are affecting her or him. I think that understanding needs to be more with the mental health services. Because when we heard these voices, it is so strange and shocking for us. (C, P11, Asian)	• People have come to visit me in hospital and brought me the Quran in English and said, Read this [name removed]. This will make you better. I think, No! I need an antipsychotic actually. It slows down my thoughts. It makes me a lot better and my food and drink. They seem to think oh just prayer and Allah's way that if you just go to the Mosque or just pray a bit more that will make you better. (SU, P1, Black British)
• When it comes to like, I'd say the Asian background, they have two sort of theories. Is it mental health or is it that somebody's done something to me? That's something that comes up quite a lot, because they don't want to accept the fact that they've got mental health issues, or they don't have an insight of mental health. And they look at it that maybe there's somebody that has done something. Even though doctors have sort of said right these are the reasons, this is what's	• …of the Bangladeshi population, is they believe in a lot of Jinn, as all these voices, all these psychotic phenomena. So, if you are not very well-versed or well-used to them, and they come in for an assessment, they describe what they see as jinn. So: the Jinn are talking to me, the Jinn are moving, the Jinn are doing this. And sometimes, if you try to explain, take the medication, do ABCD and the Jinn will disappear, they don't get it. (Occupational therapist, P14, Black British African)	• However, other people from other places - say from Nigeria - could present. Then when you try to interpret their symptoms based on what the UK practice is, or the Western style of medicine, they will disagree with you. They will tell you, 'I am not unwell, doctor, because in my culture we believe this, we believe that.' (Psychiatrist, P17, Black African)	• But there is a religious aspect to it as well. There are certain things which they believe that because of their religious notions or religious inclinations the service provider won't be able to understand, and this can be easily misunderstood and in certain cases it can be taken actually as a part of their illness as well whereas it might be culturally more appropriate. (Psychiatrist, P3, Pakistani)

happened, but they still go down looking for other answers, a cure so. (Support worker, P13, Indian)	• **<u>Refutational view:</u>** Having said that, there's also been someone that we actually saw who's from some Eastern European country, who has also had a difficulty in how he perceives his condition. So, what I'm saying is, there is an ethic and culture aspect to it. But it is one as in with any person who has their own culture, so I don't want to say it's only due to that. (Consultant psychiatrist, P9, Chinese)		
**Sub-theme: Mental health literacy**			
• I didn't know I had self-esteem issues. I had a very traumatic upbringing, and a lot of trauma had happened in my life and that was never dealt with. So, I just kept that all in my head, and I just used to lie to myself and ignore pain and I used to use alcohol and cannabis as a getaway to escape. (SU, P22, Somalian)	• Some people don't even know where to turn to in the first place genuinely and some people will know but they will be scared to, and some people will want to and will know something. (SU, P2, British Bangladeshi)		• I think it was all really new to us. The only other person that had experienced mental health issues in my family was my Grandad. My Grandad has been here as well, but I do not know if he had it over time. I think it was a bit of ignorance of what was out there. (SU, P17, British Afro-Caribbean)
• And I'm thinking of one particular person, and their family they have pharmacists in their family, and they were the people… in fact I had a GP trainee sometime back and their brother had symptoms for some time, and they just didn't, again, lack of understanding, and I'm just thinking you are someone who probably knows a bit more about this and it's still taking you some time and I don't know whether there's certain avoidance as well. (Consultant psychiatrist, P8, British Asian)	• I think particularly older, non-English speaking patients. Because they don't see, they probably don't see their difficulties as being within the mental health, you know, definition. (Psychiatrist, P9, Chinese)	• For example, Black men tend to end up as inpatients, almost a jump from seemingly well to suddenly inpatient and getting that psychological support seems to get missed in the middle and I don't know if some of that is around cultural understanding of mental health really. I think there is probably an expectation that kind of black men in particular are strong and tough and you know don't talk about emotions. (Clinical psychologist, P21, Mixed White and Black Caribbean)	• In most of the ethnic minorities, if you look into that you will come across that they have no idea that there is anything called psychology. So, the talking therapies or even the counselling, the concept of that is really pretty much non-existent in some of the minorities. (Psychiatrist, P3, Pakistani)
**Sub-theme: Cultural stigma**			
• I was finding it very hard to accept, very hard to face the people who know. I was absolutely petrified. I thought, "oh my god, what are people going to think of me, that I'm a mental case, that I'm mad. Yes, I do think that the Asian community or minority community do not get the help the help and support they should get and it's not a problem from the NHS but also a cultural issue. (SU, P18, British Pakistani)	• A lot of people don't believe in mental health, they feel it's taboo, there's obviously concerns about disclosing mental health issues to some ethnic minority families and it can be very challenging to try and get some help in the first place for some people, especially given the environment they live in. It's just that some people see it as a very sensitive subject to talk about and whilst everyone has autonomy it takes a lot of courage to go and approach people and to not have that kind of link. (SU., P2, Bangladeshi British)	• The stigma attached to mental health and especially so, and again gender (04:22) inequality and I find I'm right (04:28) a female I just brush it under the carpet and that's it we don't speak of it again. (SU, P8, British Pakistani)	• Initially, the new incidents, the person who's ill doesn't really know it. They don't want services. Because of the Arabic and Muslim background, I think there is 100% much more of a stigma for mental health, than in the White community. (C, P20, Arabic British)
• From my experiences, being from Black African household, I know that has always been the culture and it has always been the sort of stigma. We try and do our best until we really cannot. (Community psychiatric nurse, P5, Black African)	• I mention stigma because it is also something that I often hear, that other people in the community might reject or have their negative views of someone who is in contact with services and receiving mental health treatment. (Consultant perinatal psychiatrist, P3, White Mixed)	• In comparison to say White populations, I find that South-Asians and Black Africans… I find that their level of openness with regards to their mental health is a lot lower, I think it's probably because in their communities there's still a lot of stigma. (Assistant psychologist, P18, Mixed White and Black Caribbean)	• I found by working with the trust families and service users that the stigma was more painful than the actual illness itself. Coming from those backgrounds I found, that even though they were getting discharged, they were still relapsing or coming back because there was no support out there. (Mental health recovery worker, P6, Pakistani)
**SERVICE LEVEL BARRIERS**			

**Sub-theme: Dominance of mono-cultural model**			
• Because I was saying I don't remember my sister's date of birth… and she would say "are you her real sister?" I said yes, I am. She was smiling, saying that I don't even know my own sister's date of birth. It's not because I'm not interested or anything, it's because its cultural difference. (C, P19, British Pakistani)	• I still remember he was saying oh it's your life you need to avoid the kids and wife you look after yourself, you have to think about … but I personally feel like when a cycle of this they need to understand about the value of family, value or whose religion, whose background because I just feel like when they are talking to a White person they know how to treat, and one (08:30) they have to because our culture is not the same, do you understand? (SU, P20, Asian British)	• To be honest, they don't actually ask any necessary questions that they need to. They should ask a bit about how you grew up, your background, your actual history, and how it is, rather than just judge on how we look and behave, to be honest, [name removed]. (SU, P2, Black)	• I feel like if it was, just say a Black Caribbean member of staff I had, then they would've understood that why (08:56) with my dad were affecting me so much because it's quite common, to put your parents first but just the contributions I had to make to my household, it's common but a lot of people don't understand that and why it was causing me stress. (SU, P13, Black British Caribbean)<br>• **Refutational view:** I think possibly, but they were open to learning about it, which was nice. They'd ask questions, if they didn't understand they'd ask more questions to find out how best to help me and other people, so it was good. (SU, P16, Black British)
• Communication wise, if I can speak their language, I will speak to them in their language because that's one big barrier we have, because we don't have that many people in our team that are sort of, that can speak different languages shall we say. So that's one of the barriers that we have is the communication with… yeah, with some people. (Support worker, P13, Indian)	• The Black communities have expressed quite strongly that they like using the tree of life model to look at change. What's happened to them, making sense of emotional distress, don't talk about mental illness particularly but yet tree of life is not an IAPT therapy it's all based on what I presume White middle class people having their CBT being very good at doing homework. (Commissioner, P12, White British)<br>• **Refutational view:** But then you've got some that obviously don't speak the language, so we do have staff members available, we're quite a mixed team. So, we have quite a lot of members here, who are from different backgrounds, which really helps in the service. So, for example, if we do get someone, as in a Bangladeshi ethnicity, then we'll always have somebody there to translate for us. (Team leader, P8, Asian Mixed)	• Again, if you look at the general makeup of the team, I can probably count… I think we've probably only got two workers from ethnic minority backgrounds. And I think, sometimes, that can be a factor in relation to people accessing services. (Recovery coordinator, P4, White Other)	• This is different for different groups of people but generally people find it easier to have a you have X, you will be given Y, and this will lead to Z, kind of approach to stuff. It's partly the differences in how cultures look at different types of wellbeing and what that means and language barriers. When I say language barrier, I don't always mean like people don't speak the language, but I mean that there isn't always a direct translation. (Clinical psychologist, P4, Black British)
**Sub-theme: Racial prejudice and discrimination**			
• Whilst at the mental ward I did experience the staff and the care staff that were… if English wasn't their first language you would have certain patients actually verbally abuse them because they came from a different country, different continent. If there was any kind of racial discrimination or racial offences it was definitely some of the patients towards the staff there. (SU, P21, British Caribbean)<br>• What I felt was… I think because he was a Black youth, a Black male, I think the assumption was that 'oh, this is a drug user.' (C, P11, Black African)<br>• **Refutational view:** No, nothing was racist. Not from my experience. Not racially though, nothing it's not just race, so (12:36) with listening, once	• It was, yes, basically, I had issues of racism, because one of the consultant psychiatrists started saying that 'Oh, doesn't your religion feature forgiveness?', that sort of stuff. I was…I had a really nasty time with it. Really nasty time, I had, I felt I was being crucified. (SU, P4, Indian)<br>• **Refutational view:** Yeah, language barriers and discrimination because of language or multicultural discrimination, doesn't really happen because I think [area removed] is quite multicultural. (SU, P7, African)	• I said because what I think we were I feel, because of our colour they're not treating it the way they should. They have to ring the police for her, and report to the police. And were about they didn't do that to the other. They're that they had fight together, which is not fair. And this is why I say I think is because of the colour. (C, P13, African)<br>• **Refutational view:** There's no racist, no prejudice, I didn't feel like… I just felt… if the hospital took me seriously in the first place it wouldn't have got so bad. (SU, P3, British Pakistani)	• I think a lot of people just think I'm being over the top and I feel like that stereotype that we're over the top, that I don't feel like I got taken seriously and the reason I use this as an example is my partner, he works in mental health. We both got given the same diagnosis and there was a massive difference with him being a White British male in how we were treated. (SU, P13, Black British Caribbean)<br>• **Refutational view:** He said he was treated bad, but to be honest I felt like they were all okay. But he says he's been treated bad because they won't let him pray, if he's got his ... he used to have it on loud, the Quran recitation, he'd have it on loud and they'd tell him to stop it and turn it down. I don't

you're in there, you don't have much say… so. (SU, P20, Black African)			know, I can't say that they discriminated against him there. (C, P20, Arabic British)
• So, I think generalised assumptions can be made quite quickly, even harder I think when you've got a diagnosis of personality disorder because you can very much be wrongfully labelled as attention-seeking or aggressive and I guess that's a really good point as well. So, the Caribbean lady labelled as aggressive from the outset, I think it was the stereotypical angry Black woman rhetoric with this person and then when somebody has already made that assumption over the phone it was hard for them to change that, because she knew she was being seen as the angry Black woman. She felt hopeless with how the service could support her (Social worker, P17, British Pakistani)	• So, we're well aware of the problems that we have locally which is the sort of rather entrenched and sadly classic over-representation of Black, African and Caribbean men in the criminal justice section 136, acute admissions on section. (Commissioner, P12, White British)	• Unfortunately, we did have some incidences of racism as well in the team when I started. So, there was a girl who started the same day as me and she was black, she was also from Ireland. So obviously we both started on the same day. We both had curly hair, like all this kind of stuff, and I remember very vividly someone asking her where she was from, and she said Ireland. And they were like, no but where are you really from? (Assistant psychologist, P20, Irish)	• I think I was surprised and a little concerned about the difference in sectioning for people from ethnic minorities compared to people who were from a White background. It seemed that sectioning happened faster. And there was a definite disparity about discharge. Again, Black males, but not always actually. I found that with South Asian males and females, it seemed to be people were staying in care for longer periods of time than probably they should've been, and certainly compared to people from a White background. (Founder of community interest group, P2, White British)
**Sub-theme: Negative clinical interactions**			
• I remember saying I could hear noises in my attic or something like that, and she quite sarcastically turned around and said "well, do you know what a fox sounds like?" It wasn't a fox, it was auditory hallucinations, so I thought for an AMH nurse to turn around very flippantly and say, "well do you know what a fox sounds like?" - I thought "Oh my god, you're a horrible person." (SU, P14, Indian)	• They put me with a man and individuals are not trained to deal properly with certain people like the service provider said to me shake it off. How do you shake off mental health? I'm not a dog where I'm going to shake the wardrobe of my fur. It's not something you shake off; you deal with it. (SU, P2 British Bangladeshi)	• I was looking at somebody who was totally detached from… obviously, probably, a brilliant technician in his field, but people skills I didn't see. And that left the care coordinator a bit perplexed, but utter frustration because to be asked, almost like with hand movements, can you wait outside and… very much, I would use the word disdain I suppose (SU, P9, Euro-Caribbean)	• I think actually, sometimes you feel very, I don't know, there is just a judgement, it's not nice at all, sometimes sort of like when [name removed] an inpatient, you feel a bit of a judgement from, I don't know if it's the culture amongst the staff, I feel like it's maybe a culture amongst the organisation maybe, like you feel a bit judged at times and a bit like, you're not doing enough or something or you do feel that you get that sort of, like a feeling of an attitude from the staff. (C, P22, Mixed Black and White Caribbean)<br>• <u>**Refutational view:**</u> I thought the services were very good. Very good. When I got depressed and my doctor said I think you need a short stay in hospital and I was hallucinating and having paranoid thoughts, there was always a bed available. (SU, P1, Black British)
**Sub-Theme: NHS Landscape**			
	• If they went through the different areas of your life that impact your mental health and wellbeing, and say you know, would this kind of support be helpful or who's the best person to speak to? Who is your support network? Do they need support? But it's more like, sometimes you feel like you're on the receiving end of if it's not broken, don't fix it. Because if they probe and ask, then they've got more workload and it's already oversubscribed. (C, P15, Indian)	• You know you've had a few years of psychology, clinical psychology, so your quota's gone. Unless you move area and move doctor and so on, because often there is… the way in which these areas and trusts are set up is based on geography. Now how many people move from one area to another? Where does that fit in with the access of all the services that are available? (SU, P9, Euro-Caribbean)	• And I think the reason I couldn't go back was not because of Dr [name removed] being awkward or anything, I think there's just no funding in the mental health system. It is what it is, and I feel like just waiting around for the NHS, to get to the place where you need to be, because you're on a waiting list for forever is just… there's no point if you can go privately. (SU, P5, Indian Heritage)
• And that, I suppose, faith people understand, or local communities have things that are there that are supportive and are supported to do that. I suppose the question would be how that works, who funds that. I suppose it's a commissioning	• I'm sure that there would be a good role for some input on it. I think it's difficult because I don't want to advocate the NHS has maybe it's around seeing someone from a different culture, as opposed to, you know addressing specific cultures,	• I mean about 15 years ago there used to be these visits to the mosque that the mosque used to arrange with the council, where professionals could go into the mosque, have a look… You know, we used to have them periodically, I think once	• I made referrals to the transcultural team, and in a period of over more than a year, none of my patients were accepted or you could say I didn't get any response from them. However, I got indirect responses from the other resources

problem, isn't it? Because it's who…what do commissioners think they're commissioned to do, and they've got narrow commissioning remits, which means that when you think about what services need, it doesn't fit, because commissioners aren't interested in doing that, and it's not part of their remit, so…it does need a bit of a big rethink. And mental health services certainly do, because I think they're…they're not…they're really far from…they just haven't moved into the twenty-first century in terms of the provision. (Clinical psychologist, P3, White British)	but I think that would be useful if there is some more… that equals... so I suppose yes, some more training in that. But again, I don't want to go every three months with somebody, you know, it's got to be done in a way which is mindful of how busy NHS staff are. (Consultant psychiatrist, P9, Chinese)	every few months. You'd have a chat from the Imam, and just talk a bit about religious awareness, cultural awareness. I feel that used to be there so many years ago, but I guess with cutbacks and pressures and the complexity now has all changed, even pre-pandemic. (Social worker, P6, British Asian)	stating that they are really overworked, there are way less people available to provide the support. So basically, this service was not able to work effectively within the community setting at all. They were getting referrals, but the problem was there were not many people at the other end to process it and provide and the relevant support as well. (Consultant psychiatrist, P3, British Pakistani)
**Sub-theme: Tokenistic/superficial attempts to reduce inequalities**			
	• This is happening and it's been happening immemorial, and they haven't ever thought, it's 2021 they're still thinking about this and still is being talked about. It's not being manifested, it's talk the talk, if you're going to talk you must deliver. (SU, P2, British Bangladeshi)	• So, I thought they could support us a bit more if Black lives really mattered. Because it sounds daft, but the [hospital name removed], that's something they're going to be thinking about…(SU, P2, Black)	• It's not being done for us for our benefit. At least that's what it feels like anyway, I feel like there's a lot of talk. Yeah. But in terms of action... (SU, P19, Black British)
• Every time I hear that they want to make a difference, they want a more diverse people involved in the organisation, and that is from an employment perspective. Surely, we should be having people who have been through the mill that should be representing their culture, their background, to be in the forefront of what is happening. That has not been done at all, and I do not understand why. I really do not. I am hearing one message saying "yes, we are doing everything we can", and then I am seeing things that are completely different from the message that they are delivering. (Deputy manager, P1, Black British)	• The rest are all White. The NHS is sending us a continuous message that Black lives matter or other lives matter but actually they're not showing that in any of their transformation. (Charity founder, P21, Black African)	• I think if trusts and NHS generally are serious about making changes to accessing healthcare for ethnic minorities, and improving the work environment for BAME colleagues, I don't think that an exercise, a tick box activity really captures the nuances of the experiences these people go through. (Assistant psychologist, P18, Mixed White and Black Caribbean)	• I mean, I've spoken to colleagues as well, I was quite close with a psychologist who was the chair of what was originally the BME staff network, but you know, became the sort of diversity network. He shared with me that he felt very frustrated that a lot of stuff that comes from the leadership, like the board, feels to him like not actually going to make a difference, you know, and a bit of a fudge, paying lip service. Even though people might… That might be doing a disservice to some of my colleagues, you know, they do actually feel passionate, but maybe it's just very hard to make… (Consultant Psychiatrist, P7, White British)
**Sub-theme: Reluctance to acknowledge ethnicity, racism and mental illness**			
		• It's because they didn't really accept the fact of it, that some people even if they are support workers, care-coordinators, psychiatrists, they don't accept in the mental health system that people still are racist and that people are discriminatory, as well. It's out in the world, it's probably always going to be there. It'd just be nice for them to say, 'Yeah, you know what, actually you know what, he was racist, you were right about it.' It's not like that… It's all ignored, nobody is bothered about it. (SU, P2, Black)	• And it was just like "yeah" because I feel like there was nothing she could add, bless her, but that's one of the things why, when it comes to my racial struggles, I don't bring it up. I'm always trying to water it down, lie and say it's something else, but really it is about race because I know I'm not going to be understood anyway and I feel like if it was, just say a Black Caribbean member of staff I had, then they would've understood (SU, P13, Black British Caribbean)

• The privilege of White members of staff, it's not something that we're talking about, we're not comfortable talking about it. I think the Let's Talk sessions have helped a bit, but there's still a lot of defensiveness. It just reflects society in general. Society in general is still quite defensive around this stuff (Social worker, P16, White British)	• We need an apology. We need these trusts to stop abusing us and to stop being so hostile. To gaslight when we raise complaints for example. To really listen to us with race complaints instead of shoving to PALS all the time which is so antiquated and not friendly and really complex. (Charity founder, P21, Black African)	• Back when slavery was still… before slavery was abolished, how mental health disorders were made to explain behaviours that went against the rule of slavery at the time, so inherently that… I believe that trickles down into modern day - that inheritance of trauma and those kind of issues, I think it's something that's not spoken about. It's so easily put on those communities for a little bit of self-blame, it's blamed on them for not opening up then things can't get better, when in reality we're not looking at it in the lens, well this is this system made through these processes, so it is going to be hard for people to open up in those settings. (Assistant psychologist, P18, Mixed White and Black Caribbean)	• When I've trained and when I've had colleagues, that's been stuff that White health professionals traditionally find really difficult to ask, because people don't want to offend, people don't want to harm. People often say to me, I don't want to make the difference really apparent. The difference is there. The service user that you're working with knows that you know that they're Black. That's not going to change. (Clinical psychologist, P4, Black British)
**SOCIETAL FACTORS**			
**Socio-economic inequalities**			
	It has, it's been a rollercoaster because my main issues was lots, was poverty, unemployment, bad social housing and all these impacted on my social health as well as having to deal with grief and loss. I think I didn't cry straight away. Most of the tears was at night when I was by myself, just crying to myself, crying myself to sleep, trying to deal with social housing, which is very, very, very poor quality and having to deal with unemployment, having no money and having all these like priorities. (SU, P1, Caribbean)	Because I wear not very expensive clothing and all, and because I wear jewelry, as well. So, I know exactly for a fact it's discrimination, and I apologise because in the [area name removed], the fact is its known for it's racism, as well. (SU, P2, Black)	• So, I was from a kind of, so pretty deprived area, but got to this school where everybody was much more, their family's more affluent, mum worked part-time, and kids were dropped off at school in their cars. So, I think there may have been some prejudicial overshadowing there that meant that the clear difficulties that I had emotionally were misinterpreted, dismissed, ignored, resented. (SU, P19, Black British)
• I think that honestly most of the issues that induces mental health problems in ethnic minorities could be avoided if those were addressed, if housing was addressed, if access to medical care was addressed. I work with people who for years have been denied healthcare, these are people with zero recourse of public funds. You are in the asylum process, or you are undocumented. You have no access to healthcare, even if you have a cough, you self-medicate because you are not allowed to access a GP. At the same time, you do not have a job, you are homeless. All of those issues, they are just impacting on your mental health. (Charity founder, P6, Black African)	• Well I'm not sure to be honest, but yes, some of the most vulnerable people in my service still come from ethnic minority groups. And by most vulnerable, I mean people have recently moved to this country, don't know the system, don't have much support, might be isolated, more in a relationship that is characterised by domestic violence. They may not know the language or have financial resources, they may not be independent, so in a way being ethnic minority group it is part of the picture. (Consultant perinatal psychiatrist, P3, White Other)	I know the client who is other European actually had, they were classed as shame status, so they were homeless, and they were able to obtain shame status within the UK. So, they've managed to now get a British passport and also be on benefits. I think a lot of the challenges have been in relation to getting on benefits, receiving an income, which obviously has a wider impact. (Recovery coordinator, P4, White Other)	• It's really socio-economic as well, which then of course goes hand in hand with your psychological issues as well. Of course, if you are not having enough money, not food on your table you can't be psychologically stable all the time, even the best of us. The other complex area which I found was the refugee and the asylum seekers. This has been a really, really problematic area even before covid but in the post covid, as I said earlier, there are socio-economic factors there. (Consultant psychiatrist, P3, British Pakistani)
**Systemic racism**			
	• No, I have had really appalling practices, I must admit, and I can't blame just the mental health,	Oh, this is what it is, and this is why I basically keep myself to myself, because I know for a fact of it, I'm	• And also, I'm not entirely sure about this, but in terms of employing people, I do have some

	it's the GP's, it's the school. Because that discrimination started for my son from school I was asking from school for the right diagnosis, and the right care and support and it never happened. (C, P19, Asian British)	Black. I know I sound Jamaican as well, because you can hear it in my voice. I know for a fact of it…. (SU, P2, Black)	suspicions that sometimes it can be due to their race, even with the Discrimination Act, all of that, I still think there can be situations where… it can't be just in the NHS, but generally employers might not employ someone based on their ethnic background and those sort of issues and other reasons. I'm not 100% sure, it's just an assumption, just generally, I don't think it's just in the NHS. (SU, P5, Indian Heritage)
• I think it's also difficult when somebody has had a significant experiences of hate crime, or a racial abuse within services or within the wider world. And that is then kind of perpetuated in a cycle of anxiety for example or avoidance. (Occupational therapist, P4, White British)	• Well, all of our clients are black and brown citizens. Our experience of working with our clients across [area names removed] is that they are facing the same intersectional challenges, both with mental health services. So just really a range and all of it very much linking back to structural racism, the lack of anti-racism pathways, the lack of understanding how systems are really at the moment under duress because of austerity and other issues. (Charity founder, P21, Black African)		• We had a community development worker, but we actually didn't have a lot of ethnic diversity in the team, and that becomes another big challenge that people are bit awkward about. Again, it's a big sort of structural thing in society, but then you see a lot more ethnic minorities at lower pay grades. (Consultant psychiatrist, P7, White British)

Supplementary Table 6. Covid-19 impacts and negative and positive developments: subthemes and illustrative quotations organised by site and participant type (service user/carer followed by professional; ethnicities are self-reported; interviews conducted across four sites covered by NHS trusts: Coventry and Warwickshire; East London; Greater Manchester; Sheffield)

SITE 1	SITE 2	SITE 3	SITE 4
**COVID-19 IMPACTS**			
**Sub-theme: Reduction in access & regularity of mental health services**			
• I felt that they completely went silent. Completely silent. It would have been a blessing if somebody rings you just asks you how you are, how you're coping with your sister's situation, or how your sister is coping with the mental health, because there are so many (27:01) that cannot actually come out and do anything about you. (C, P19,	• So, during Covid if I needed help, I would have to seek it out myself. My care co-ordinator or my team weren't calling me; I was the one that had to seek help if anything happened. (SU, P7, African)	• It's just that not much has been able to be done as it used to be with the pandemic there, to be honest. (SU, P2, Black)	• So no, I think before Covid it was bad, that was my experience of it but afterwards it was worse which I understand. (SU, P13, Black British Caribbean)

British Pakistani)			
• So, from the crisis team perspective yes, lower level of engagement to the point where we were meeting people of ethnic minorities at the stage that it was Mental Health Act assessment or hospital admission or crisis home treatment team because they weren't able to seek help at the preventative level. So, I think yeah engagement has potentially reduced. (Social worker, P17, British Pakistani)	• No. I think there has been an effect more on certain groups as well. From our results we're seeing it and we're trying hard to put more emphasis and more focus into particular groups. So, the ones that we've found affected, again varies depending on what's going on. But we are finding a vicious drop out of minority groups, especially from the Black community, Black Caribbean community. They have really shot down in the numbers. Especially in [area removed] which has a large amount of, the community are Black and Caribbean. (Service manager, P18, White British)	• Since COVID it has been a lot more difficult getting translators at very short notice, so I've had to cancel my patients' appointments. Over the last couple of months, I've cancelled about three or four patients' appointments because we couldn't get translators, they couldn't communicate with me. The major problem - to answer your question - has always been communication problems. (Psychiatrist, P17, Black African)	• And again, all these things get complex but their pre-existing ability to access the service and the services to provide the care as well. So, if it was already compromised then I have noted that it became more compromised. Especially the poor social interaction. This generally affected everybody irrespective of the ethnic race and everything. But I think it became more noticeable for them because it was already a lack of resources for them. And they were left pretty much really in a very difficult situation there. (Psychiatrist, P3, Pakistani)
**Sub-theme: Remote delivery of mental health services**			
• Because I was better mentally at the time, like I was recovered, I would say there was no difference to me, but I can imagine for someone who was struggling and who hadn't recovered, a face-to-face would be the best thing for them, and that's just because the contact you get when you speak to someone on the phone is not the same as the experience you get in person. It's a bit more personal and they can see like how you react to things, and it's just in general more personal and more engaging. (SU, P10, Black African)	• 100%. The third country people that are ethnic minorities they would. For me it was okay to do online, on telephone and things like that. A lot of people won't have that comparison because they might be very lay, they might not be able to use a phone properly, they may not be able to speak the language properly, they may not be able to engage with the technology but for me that wasn't an issue (SU, P2, British Bangladeshi).<br>• **Refutational view:** Which is not bad for me, when virtual is more accessible to me because due to my mobility issues. Some of the places, therapies, are in a building that do not have a lift or are difficult to get transport and to organise. (SU, P6, East African)	• She can get confused with the remote consultations; that's why she prefers in-person ones. (C, P5, British Asian)<br>• **Refutational view:** Yeah, so basically, they stay on the telephone which I prefer! Yeah, rather than going to the hospital I prefer it on the telephone in the comfort of my own home. (SU, P14, Black African)	• That was one thing…I still think I would have (22:11) if I saw him face to face, because he just doesn't listen, but on the phone it's much more easy to say that to someone, and dismiss them, because you're not doing it to their face. So in terms of switching to phone calls during the pandemic, it had an impact of, just an additional thing of not being listened to, just an additional barrier. (SU, P5, Indian Heritage)<br>• **Refutational view:** I'd only just started it before the pandemic hit and before then when I first went into it, I didn't want to be seen face-to-face, so it was always phone call situations, which was more comfortable for me. (SU, P16, Black British)
• Yeah, I think the biggest thing really comes back to languages because especially during high levels of restriction the majority of our services were provided over the phone. And if you have somebody that struggles with English, and you are not able to use that body language, gestures, you know, nods and encouragement, that makes it more difficult. (Occupational therapist, P4, White British)<br>• **Refutational view:** Some people, it's easier for them to do it from home, to have friends interpret for them and family on the sofa, they haven't got to travel halfway across town to get to a mental health appointment, to CMHT…so yeah, I think a bit of both.	• But obviously, when you are assessing, you have to rely on what they say. So, when the client starts… their English is limited… I think we saw that sometimes we missed things out. Not major things. But I think sometimes, before, we knew they were much more distressed than they normally would be, just because you don't see that face-to-face and… they can't express themselves openly. (Occupational therapist, P14, Black British)<br>• **Refutational view:** In a way, because of the nature of what we deal with, this can be seen as, again, a very shaming, stigmatising… and speaking on the phone is easier than being… So, I can see where they're coming from, as well. (Consultant Psychiatrist, P11, British Indian)	• And I don't think it was a productive way for these people to engage they need face-to-face. They need that relationship and trust. It's hard enough when you can't fully understand somebody talking to you and what they're explaining. We could say something where we'd go, 'Do you want Subutex?' And they'd be like, 'We don't understand.' But then we'd be stuck because there'd be no other way to explain it to them. (Recovery Coordinator, P1, White)	• And then how you engage with looking at a screen were issues across the board, but certainly within people from a South Asian background particularly, that was a real struggle. It impacted the sense of how do you connect with this White person that's sit… because they don't know who I am and I'm having to explain through a screen. So, that physical barrier really hampered it. (Founder of Community Interest Company, P2, White)<br>• **Refutational view:** But on the other hand it's giving them the opportunity as well. So, if the clients are not at home, they receive a phone call from the doctor which they can attend anywhere. Yes, so as I said attendance has improved with it but at the same time, I think it… at first for most of the clients it was a relief in a way. They're not time bound, they don't have to commute. So, it saves different things, your time, your finances, and those kinds of things. (Psychiatrist, P3,

			Pakistani)
**Sub-theme: Heightened risk/expectations on minority ethnic staff**			
• I felt that the staff never behave this way towards patients or to me particularly. It was because of Covid, everybody was under strain, everybody was trying to make sure the social distancing was kept, and I thought that this incident was a direct result of the Covid situation. Like I explained about my experience, I felt the staff had a lot of pressure and for that reason they weren't as attentive as they have been previously. The shortage of staff did really affect them. (SU, P18, British Pakistani)	• At first, a few was like not knowing what was going on, it was quite strange. It was like there was a lot of stress on the services, however, it was just, it was just having understanding, mutual understanding, being able to give and take and respect. Sometimes I was the one asking my services, like my care coordinator and things: "are you okay? Are you taking time off?" (SU, P7, African)	• So, I think sometimes you need CBT, you need outdoor activities, and I think you have to really push for that, or they'd be like, 'yeah, we need to do this, we're short staffed at the moment, we're making room for everyone, a doctor needs to do this, we need to review all of this. (C, P22, Pakistani)	• She went above and beyond but I feel like a lot of the staff, because of Covid, didn't make the same effort that they would've had to before. (SU, P13, Black British Caribbean)
• Yeah, but with our service, the community support service, there's only a handful of people that were willing to go and do face to face. So, myself and a couple of my colleagues were the first out of our organisation to go out, that community base, to go out and do face to face support. We started like two, three months after the first lockdown so we had started to do… Because we knew it was having a massive impact on our service users. (Support worker, P13, Indian)	• She was talking about, because she's not from this country, and what she observed was that, if we say… The local staff, not from those backgrounds, actually, we're more likely to not be as proactive or wanting to support clients with Covid or suspected Covid or going to red zones and things like that. The other thing to be aware particularly in my field is that predominantly the workforce is from Black-Asian and minority ethnic backgrounds, as well. So, it's a reflection from a colleague about how the minority workforce felt, that they felt that they had to go in, they had to go on the red zones, they had to that, whereas the others just stepped behind, even full on knowing that the risk was higher. (Consultant psychiatrist, P11, British Indian)	• I think, from my perspective, from being in the BAME community myself, was just that I was very vigilant about ensuring that I had the proper PPE, that the patients had their masks, that the patients were a suitable distance from myself. And, I have the biggest office in our building actually, so I did more than my job required to support some of my colleagues by seeing people in this room. (Recovery Coordinator, P10, Black)<br>• **Refutational view:** I'm from an ethnic background as well I was basically forced to go home. Even though I said, no, I don't want to work from home they said, you have to. You have no choice. (Recovery Coordinator, P7, British Pakistani)	• Which has happened in the NHS for a really long time, asking Black and Brown members of staff to do the jobs that people didn't really want to do, or asking them to do overtime. At one point the group of people who died, relatively was the Filipino nurses, because in Philippine culture there's a rule around saying no when people need support. They were asking these Filipino nurses to work 12-16-hour shift six days a week because they knew they wouldn't say no. Things like that. This will have a lasting impact. (Clinical psychologist, P4, Black British)
**Sub-theme: Closure of community organisations**			
• I was only disappointed with [charity name], like I said - but they're not the Trust - and I still am. The fact that they chose to close the Hub - they didn't think of an alternative method. I know that CGL, for example, were meeting out in the open but they were staying two metres apart - they thought outside the box. (SU, P14, Indian)	• It did actually, yeah, because I like to go to the Sikh place of worship…. basically, it was actually… they had to be closed because of the pandemic. (SU, P4, Indian)		• Yeah, before I go to church. Before Covid but after Covid, I can't. I didn't go because before I go to church, you must test for Covid. (SU, P11, African)
• Again, working within the community mental health team, the majority of the people that I work with rely heavily on the community organisations, not just the support they receive through the NHS. And everybody has had those things removed whether that be, attending church, or going to a mosque. Whether that be, you know, like a day centre service. Or whether that be, you know, the local Pujabi centre. No matter the community service that you access, it hasn't been there. Perhaps there is a greater reliance on these services within the minority ethnic groups but that would be a total guess. (Occupational	• We're picking up Bengali women and Somali men, but that's just a small example of… Lots of other minority groups who are kind of backing off, because they get to a stage where they need that support, and they normally get it from the community to be honest with you. And they're not, they're not getting it from their local community, not as much.' (Professional, P18, White British)	• So, we couldn't get people out to services back into their own areas where other treatment may be available - mutual aid, things like that. Because I know there is Farsi NA groups in [area removed]. And there is other cultural - I know there is Chinese NA group in [area removed] as well. But it's things like that, that unfortunately everything is just gone all at once. It's only just starting to re-open again. (Recovery Coordinator, P1, White)	• So, I think on a basic level Covid seemed to hit people in a way that maybe other communities weren't experiencing it. Again, the loss of the relational impact… for the Muslim community the loss of things like Eid and Ramadan, in terms of community practice and what that meant. (Founder of Community Interest Company, P2, White)

therapist, P4, White British)			
**Sub-theme: Media scaremongering and scapegoating**			
• Yeah. I don't watch a lot of TV, out of choice. This isn't me being a conspiracy theorist - but I think the masses are largely controlled by what they see on TV. I knew that there was a Covid pandemic because I was working in the hospitals, but I didn't sit there endlessly watching TV. When I went to my brother's house, they were watching BBC News and it was constant and relentless, and it made you have anxiety (SU, P14, Indian)	• And the news was all bad, this many deaths, this many of this and that, everything was really very doom and gloom, so it was quite a scary time, yeah. (C, P22, Pakistani)		• I really wanted a girl, one of the reasons was because of the George Floyd stuff and I was so scared having a son that even though he's mixed race, I'm aware people easily in this country can put mixed race people into the Black category so I was like, "oh my god, I'm having a Black son, these things could happen." (SU, P13, Black British Caribbean)
• Only to the point that the media and the information that has come from the media as they portrayed it. It is interesting, because there was a good point you mentioned that if you feel like you have been disproportionate… where is the evidence. When you hear something about the pandemic and how it is affecting the majority of African-Caribbeans or people from an Asian background more than our Caucasian counterparts… it is that information that concerns me, where did that come from and why would it need to be mentioned? (Deputy manager, P1, Black British)		• I think a lot of media scaremongering blaming different ethnic minorities has stopped people from socialising and engaging because of fear of persecution. Or in fact, actually in [area removed] it's not been nice because we have a massive mixture of cultures in [area removed] and it has been quite against one or the other.' (Recovery coordinator, P1, White)	• Because on the news there was stuff like, we need to put [area removed] on a local lockdown, because it's going to be Eid and all the borough people are going to be out and mixing with each other, versus my actual experience of that, which is not having seen family members and friends for months at a time. There was lots of things online and on TV essentially blaming Black and Brown people, versus what was actually going on in the Black and Brown community. (Clinical psychologist, P4, Black British)<br>• That instant more anxiety, more fear, you can't unpick that from George Floyd's murder, because that was all happening at the same time, and it felt really intwined. (Clinical psychologist, P4, Black British)
**NEGATIVE DEVELOPMENTS FROM COVID-19**			
**Sub-theme: Scepticism and distrust**			
• An inside note, what I found strange in watching TV and especially with supporting NHS staff and seeing how tremendously they have worked and supported people in this pandemic. My experience at the hospital was totally different. The hospital was almost empty. (SU, P18, British Pakistani)		• What happened was, it, they gave me my first vaccination, and between my first and second vaccination I had an issue with the, my mental health. And they kept sending me messages saying, 'go and get your second vaccination' and I started thinking that I don't need my second vaccination. And I only got it yesterday, so I had my second vaccination about a year and a half after my first one. So that's, I guess, down to my own mental health. (SU, P15, Asian)	• We've had the issues with the gas in the air. People with Corona got heart problems, they start dying and getting worse and worse. That's what kicked off…. Young. 59, I think. The old ladies, they died two days after they got boosted. (SU, P12, Pakistani)
• When COVID -19 started, there was a whole ho ha WhatsApp community sending and spreading false information. So obviously there's that whole mistrust in general not just for mental health. That whole mistrust of ethnic minorities in NHS professionals or sort of "what is it that they want - oh my goodness they better stay away from me." (Community Psychiatric Nurse, P5, Black African)	• So, before pandemic, during pandemic, it's still the same thing, because, I've said, with mental health, there is a lot of paranoia around it, coming when working with people. We had someone not so long ago, where he was from the Bangladeshi community, and he thought it was all a plan to get rid of all the people that have mental health. (Team Leader, P8, Asian Mixed)	No trust. So, before the pandemic the patients used to come in engage well. But once the pandemic hit, obviously in the ethnic community background there was a lot of false news going about. Like don't have the COVID vaccine, the government are just out to kill you, do not go to the hospital whatever you do, you won't come out alive. So, it did create a very, very big panic in the community. (Recovery Coordinator, P7, British Pakistani)	• There's lots of anxiety, lots of worry, lots of distrust and also anger. People had not left their home for months. People were doing everything that they could to protect themselves and their families and every time we turn the news on there was some question around whether Eid was going to be the thing that tips of edge. It's just horrible. (Clinical Psychologist, P4, Black British)
**Sub-theme: Anger, frustration and disillusionment**			

• I think because every time you're trying to (24:53) they will say, oh its corona, we can't do much, you have to try your best to manage it. But because of the pandemic they can't do much about it? But it was frustrating when you really need or you're desperate for the help. But they say there is nothing much we can do. (C, P19, British Pakistani)	• I say it was a bit frustrating at times because the level of the cases and the care coordinators and the team actually escalated, and it was a lot more so sometimes you would be calling in the office and the lines would be busy, so it was quite frustrating but then it actually let me deal with things. (SU, P7, African)	• No, it's just in general... just in general that changes psychiatrist left right and centre, and I think really that sort of ill person that myself, you need at least a psychiatrist who's going to be a psychiatrist. You don't like explaining the story again and again. That relationship with a doctor trusting. You talk to him and then you're gone and it's the same again really. You get quite sick of it, to be honest with you. (SU, P10, Pakistani)	
• Perhaps a reflection on the health inequality that already exists within our system, and I guess upset, resentment and anger around that. (Occupational Therapist, P4, White British)	• I personally got during that period much more angry. I was angry. I was angry at decision makers, angry at services because they had the money. Eventually the central government pumped in millions to prop up structures, but it was just not filtering down to minoritised communities across any service. So, I was very angry last year more than happy. (Charity Founder, P21, Black African)	• Yeah, I think it was a lot of anger and, like I said, a lot of the content of conversations changed from being recovery focused and what they were before to being more a space for people to vent, because it was an incredibly difficult time. I think there was a lot of anger about the mixed messages that were given from the government, especially when simultaneously you had MPs going out and driving miles to check their vision and things like that. (Assistant Psychologist, P18, Mixed White and Black Caribbean)	• So, I think on a basic level COVID seemed to hit people in a way that maybe other communities weren't experiencing it. I think there is a sense that some communities, particularly I think the Black community, the Black Caribbean community feel quite left behind and so there was quite a lot of anger and that led to some psychological feelings of isolation and of loss. (Founder of Community Interest Company, P2, White British)
**POSITIVE DEVELOPMENTS FROM COVID-19**			
**Sub-theme: Empowerment**			
	• But it's only like recently like during Covid, I decided to act upon my side effects because I thought to myself, I'm not… Thank God, you know I'm still living. Even during Covid I haven't had any Covid reaction, and I thought I'm going to tackle my side effects because I'm stable and I want to improve my… I just want to improve my flow and my… I want to improve my body. I started to go to the gym, and I was thinking I just want to just be all round internally fit. (SU, P1, Caribbean)<br>• Because I'm a writer, I do like creativity to deal with the pain and anguish I was going through. And that allowed me to release all the inner feelings, negative feelings that was getting me inside, into culture, (08:25) play or prose. And that got me through the journey. But it was very difficult. But I was able to kind of thrive on that level. So I've been trying to eat good food… Go out for walks and have a coffee and just take a bit fresh air, and just release the pain that's going on, and like I said with creativity, it's helped me to write the best poems I've ever written, I think, yes. (SU, P5, African)		
	• No, I think… some of the service users, actually… the resilience that they showed to a number of us – and I'm talking about people who've been working in mental health for 25, 30 years,	• And I also think with a number of them it was a bit empowering as well, we were having some model, I mentioned before the maps that we do in the therapy that I offer and normally I'd draw	

	sometimes 15, 20 years – none of us ever thought that clients who were basically on our doorstep every single day could actually cope with just a phone call, or limited… But they were amazing! So, I think that's something that we learnt, actually. And I think it's actually made us know… maybe the system of care that we have provided, from a recovery perspective, we need to push the strengths-based model much more. These people, they have strength that we need to… work with them when it comes to their care plans, recovery, whatever it is. (Occupational therapist, 14, Black British)	those out on a paper on a table between and obviously I couldn't do that online. But young people are creative, so they'd create the map and share it with me and so for them they were really taking ownership over their therapy. That felt really positive, that they were able to do that. (Clinical psychologist, P21, Black and White Caribbean)	
**Sub-theme: Flexible approach to service provision**			
• I've found it easier to be honest. At the start it was harder but now, I can get a hold of people much easier. Sometimes having these kinds of video calls helped me. (SU, P12, Black African)	• I think… they've still had to make improvements and adapt in other areas of health because of Covid, because now when you ring the surgery, the GP practice, you can have a phone consultation instead of having to go all the way in the surgery. And sometimes that's all you need. I think because they have to adapt now to the Covid pandemic, they're trying to make other areas of health more accessible. I would say they've made it more accessible. (SU, P1, Caribbean)		
• So, I think we have to be more tech savvy, so that we engage with people in a different way. That we use our IT in a different way that people can just reach out and get some help online. Because some of the things are going to be, like they're factual isn't it, so I just feel that all these apps that we have these days, we should be using more of that so that people can just dip in and out very easily rather than worry about seeing someone. It should be a smoother or easier process to maybe just get on your computer or your phone and try and understand what's going on. (Consultant psychiatrist, P8, British Asian)	• I think it's also given us new ideas as to how we move forward in terms of care provision – in terms of how we deliver care. Can we… we are slowly moving back to what we used to be. And we have made a decision here: it needs to be mixed; it needs to be a hybrid model. So, we are thinking of this in terms of… Groups, for example. Groups used to be purely face to face. But now they do groups online. We have online groups for ABCD, and we have hybrid groups. It's got us talking about it…(Occupational therapist, P14, Black British African)	• But I think some of that was about no privacy within the family home and so we ended up kind of having like an email therapy really, felt like the safest option. Quite unusual definitely, not something I would've explored pre-Covid, but it worked, and it worked really, really well. One of the things that did come of Covid is I do think we learned to be a lot more creative and accepting good enough. I think it was definitely a good thing rather than always wanting things to be perfect. (Clinical psychologist, P21, Mixed White and Black Caribbean,)	• I'm quite a prime example of that myself actually, how it has affected us. So, I have always seen patients face to face. I never had the concept actually it can be done online. But now I'm talking to you online the same way I talk to my clients online as well. This is a big change. For both it has its own pros and cons as well. Yes, I just thinking actually sometime back is one of the good things was it improved attendances actually of the many clients. There are less DNAs. (Psychiatrist, P3, British Pakistani)
**Sub-theme: Spotlight on inequalities**			
	• Yes, and you know when I talked about institutional racism outright, they just don't want to believe it, but there is injustice, inequality for the BME community, now they are picking it up with the pandemic it's certainly been picked up hasn't it? More visual. (Carer, P19, Asian British)	• During hospital there for a reason, and they're not going to be forgetting what's happened last year, and the year before with Black Lives Really Matter and then to go with the pandemic. I know they could see people a little bit better, to be honest, if that's okay to say. (Service user, P2, Black)	
• And I also think there's been more open - there's been more conversation, there's been more conversation in the Trust about some of the issues, which is helpful, because they've talked about it more amongst the staff group, which has enabled more acknowledgement in patients. (Clinical	• We ran the UK's first digital lottery funded women's iPad mental health peer-to-peer session, Somali women. We had to buy iPads, and everyone had pads at home. Over 40 people engaged with, and it was a weekly/twice weekly session just to keep clients feeling less vulnerable. (Charity		• But there were a lot of people that were donating laptops to organisations and places, to hopefully fill those gaps and then each organisation knew their clients better, so they tried to use their ways of making sure how these people would be accessing the zoom. For example, we used to do

psychologist, P3, White British)		one-to-one coffee mornings to keep them engaged because they had mental health issues and it was the nature of their wellbeing, so it was my priority to get support for those who couldn't afford it and I did request and apply, and I managed to get 19 iPads. (Mental health recovery worker, P6, Pakistani)

Supplementary Fig 1. Process of analysis: Ma[illegible]

Familiarisation & coding

Charting

Mapping

Areas of Inquiry

Challenges for people from minority ethnic groups accessing/using MH services prior to the Covid-19 pandemic

Emerging themes

Prior to Covid-19

1. MH services too Western oriented
2. Barriers to access for people from minority ethnic groups
3. Complex proble[illegible]privation
4. Systemic disc[illegible]
5. Tokenistic initia[illegible]

Impacts of Covid-19

1. Everyone affected
2. Exacerbated disparities
3. Remote working and digital exclusion
4. Mistrust
5. Empowerment

Contextual factors

1. Regional variations
2. NHS context
3. Limited resources
4. Political backdrop

Thematic headings with index

Mental health services too Western oriented
1.1 Lack of staff diversity across levels
1.2 Lack of attention to personal stories & cultural background
1.3 Lack of culturally appropriate service provision
1.4 Limitations of the Western medical model
1.5 Inadequate training & guidance
1.6 "Alien place"
1.7 Communication barriers
1.8 Avoiding conversations about ethnicity & racism
Cultural backgrounds, norms & understandings
2.1 Cultural stigma
2.2 Explanatory models of mental illness
2.3 Alternative help seeking
2.4 Treatment preferences
2.5 Family expectations
2.6 Lack of awareness of MH and MH services
2.7 Mistrust of MH services
Racial prejudice & discrimination
3.1 Differential diagnosis, treatment & management
3.2 Preconceptions, stereotyping and assumptions
3.3 Institutional & structural racism
3.4 Tokenism & inaction
3.5 Expectations on minority ethnic staff
3.6 Intra-racial conflicts
Non-specific challenges
4.1 Delays and lack of continuity of care
4.2 Staff attitudes
4.3 Deficient communication
4.4 Over-reliance on pharmacological interventions
Reduction in availability of MH care
5.1 Reduction in regularity and quality
5.2 "Missed and forgotten"
5.3 Reduction in community supports
Changes in service delivery
6.1 Negatives impacts of remote service delivery
6.2 Positive outcomes of remote service delivery
6.3 Social distancing and PPE
Feelings towards services and authorities
7.1 Disillusionment, anger and frustration
7.2 Mistrust
Media coverage
8.1 Fear mongering
8.2 Stereotyping and scapegoating
8.3 Social media
Impact of different pandemic stages
9.1 Psychological and behavioural
9.2"All in it together" – counter code
9.3 Calm before the storm
9.4 Staff burnout
9.5 Exacerbated disparities
Positive reactions and resilience
10.1 New ways of working
10.2 Self-determination and resilience
10.3 Spotlight on racism, inequalities and MH
Contextual factors
11.1 Social disadvantage & deprivation
11.2 Regional variations
11.3 Socio-political context
11.4 NHS landscape
11.5 Austerity
11.6 Complex trauma

Challenges in mental healthcare

Racial inequalities sustained by service culture & design
1.3 [illegible]
1.2 [illegible]
1.8 [illegible] ethnicity & racism (1.8, 3.4)
3.1 Racial prejudice & discrimination (3.1, 3.2)

Cultural backgrounds, norms & understandings
2.1 Cultural stigma (2.5)
2.2 Supernatural illness attributions
2.3 Alternative help seeking
2.6 Lack of awareness of MH and MH services
2.7 Mistrust of MH services

Quality of care
4.1 Delays & lack of continuity of care
4.2 Lack of person-centred care (4.2, 4.3, 4.4)

Effects of the Covid-19 pandemic

Mental Health services
5.1 Reduction in availability & quality of MH care
6.0 Changes service delivery (6.1, 6.3)
9.5 Exacerbated disparities

Wider context
5.3 Reduction in community supports
9.1 Psychological and behavioural
8.0 Media coverage (8.1, 8.2, 8.3)

Outcomes of the Covid-19 pandemic

MH crisis
5.2 "Missed and forgotten"
9.4 Staff burnout
9.3 Calm before the storm

Altered relationships with services and authorities:
7.1 Disillusionment, anger and frustration
7.2 Mistrust

Positive reactions and outcomes
10.1 New ways of working
10.2 Self-determination and resilience
10.3 Spotlight on racism, inequalities and MH

Contextual factors

3.3 Institutional & structural racism
11.1 Social disadvantage & deprivation
11.3 Socio-political context
11.4 NHS landscape

Pre-covid 19 barriers

Individual
2.1 Cultural stigma
2.2 Supernatural illness attributions
2.6 Lack of mental health literacy
2.7 Mistrust of MH services
Service
1.3 Dominance of mono-cultural model
1.8 Superficial initiatives & reluctance to acknowledge ethnicity & racism
3.1 Racial prejudice & discrimination
4.1 NHS landscape
4.2 Negative clinical encounters
Societal
Systemic racism (3.3)
Socio-economic inequalities (11.1)

Covid-19 impacts

Reduction in access & regularity of services (5.1)
Remote service delivery (6.1, 6.2)
Heightened risk/expectations on minority ethnic staff (9.4)
Closure of community organisations (5.3)
Media scaremongering and scapegoating (8.1, 8.2, 8.3)

Outcomes of barriers and Covid-19

Communication difficulties
Disengagement
Isolation/alienation
Avoidance/delayed help seeking
Alternative help seeking

Negative developments

Scepticism/distrust (7.2)
Anger/disillusionment (7.1)

Positive developments

Empowerment & resilience (10.2)
Flexible working (10.1)
Spotlight on inequalities (10.3)
